# Genome-wide association study of salt tolerance at the seed germination stage in lettuce

**DOI:** 10.1371/journal.pone.0308818

**Published:** 2024-10-18

**Authors:** Modan K. Das, Sunchung Park, Neil D. Adhikari, Beiquan Mou

**Affiliations:** USDA-Agricultural Research Service, Sam Farr United States Crop Improvement and Protection Research Center, Salinas, CA, United States of America; Government College University Faisalabad, PAKISTAN

## Abstract

Developing lettuce varieties with salt tolerance at the seed germination stage is essential since lettuce seeds are planted half an inch deep in soil where salt levels are often highest in the salinity-affected growing regions. Greater knowledge of genetics and genomics of salt tolerance in lettuce will facilitate breeding of improved lettuce varieties with salt tolerance. Accordingly, we conducted a genome-wide association study (GWAS) in lettuce to identify marker-trait association for salt tolerance at the seed germination stage. The study involved 445 diverse lettuce accessions and 56,820 single nucleotide polymorphism (SNP) markers obtained through genotype-by-sequencing technology using lettuce reference genome version v8. GWAS using two single-locus and three multi-locus models for germination rate (GR) under salinity stress, 5 days post seeding (GR5d_S) and a salinity susceptibility index (SSI) based on GR under salinity stress and control conditions, 5 days post seeding (SSI_GR5d) revealed 10 significant SNPs on lettuce chromosomes 2, 4, and 7. The 10 SNPs were associated with five novel QTLs for salt tolerance in lettuce, explaining phenotyping variations of 5.85%, 4.38%, 4.26%, 3.77%, and 1.80%, indicating the quantitative nature of these two salt tolerance-related traits. Using the basic local alignment search tool (BLAST) within 100 Kb upstream and downstream of each of the 10 SNPs, we identified 25 salt tolerance-related putative candidate genes including four genes encoding for major transcription factors. The 10 significant salt tolerance-related SNPs and the 25 candidate genes identified in the current study will be a valuable resource for molecular marker development and marker-assisted selection for breeding lettuce varieties with improved salt tolerance at the seed germination stage.

## Introduction

Soil salinity is one of the most important abiotic stresses limiting crop production worldwide. Soil salinization can arise from natural causes, or from human activities. Natural causes include rock weathering, high temperatures, low rainfall, and closeness to salt water. Climate change further exacerbates this issue, with rising temperatures promoting increased transpiration from plants and evaporation from soil, leaving even more salt behind in soil. On the other hand, human-induced salinity results from deforestation and agricultural practices such as irrigation and chemical fertilization. Food and Agriculture Organization (FAO) recently reported that more than 833 million hectares of subsoil and 424 million hectares of topsoil are salt-affected globally (https://www.fao.org/soils-portal/data-hub/soil-maps-and-databases/global-map-of-salt-affected-soils/en/).). Therefore, many farmers worldwide grow crops in salt-affected soils. The most economic and environmentally friendly solution for managing soil salinity problem is through development of salt-tolerant crop cultivars.

Lettuce (*Lactuca sativa* L.) is one of the most important leafy vegetable crops globally and the U.S. is the second largest (by weight) lettuce producer in the world behind China. In 2022, lettuce accounted for nearly one-fifth of the $21.8 billion earned by the U.S. growers from sales of vegetables and melons, including romaine, iceberg, and leaf type lettuce sales of $1.54 billion, $1.33 billion, and $1.25 billion, respectively (https://www.ers.usda.gov/data-products/chart-gallery/gallery/chart-detail/?chartId=106516). California grows more than 70% of the U.S. lettuce, with the main producing region located in the Salinas Valley [1]. Commercial lettuce production in the irrigated valleys of the southwest, is often located on soils that are somewhat saline or at least subject to salinization and soil salinity in the Salinas Valley is becoming a growing concern especially due to its proximity to the ocean and resulting seawater intrusion [2, 3].

Lettuce is a self-pollinated annual crop species with a diploid chromosome number of 2n = 18 and a total genome size of 2.6 Gb. The wild species of lettuce, *Lactuca serriola* is known to be one of or the only direct ancestor(s) of modern-day lettuce [4–8]. Genetic diversity in shape, size, and color among lettuce cultivars exists and based on leaf shape, size and texture, head formation and stem type, it is generally classified into six horticultural types: crisphead, butterhead, romaine, leaf, stem, and Latin [8].

Germination and establishment have been identified as very important stages in crop growth and productivity, however, most studies on the effects of salt stress on plant growth have focused on stages beyond early seedling growth [9–11]. Salt stress inhibits seed germination by reducing water uptake (osmotic stress) and creating ionic imbalance within the seed (ionic stress) [11]. However, a recent study reported that lettuce seed germination is inhibited by salt-induced osmotic stress rather than ion toxicity, since seeds could germinate fully after salt solutions were removed [12]. A previous study reported that 60 mM NaCl salt stress delayed and reduced germination of lettuce seed [13]. A study on the effect of salinity (0–120 mM NaCl) and temperature (20–30°C) on germination of ‘Phoenix’ lettuce seed, reported that increasing levels of both factors inhibited germination and reduced fresh weight of young seedlings [10]. The interaction between salinity and temperature had a significant inhibitory effect on germination and while lettuce seed germination at lower salt levels (20–40 mM NaCl) was similar to the control (water) at 20°C, it was considerably reduced at 30°C [10].

The sensitivity of lettuce to salinity causes reduction in yield and affects grower profits. Lettuce seeds are planted half an inch deep in soil where salt levels are often highest in salinity-affected growing regions. Therefore, it is imperative to breed lettuce varieties that can germinate and establish in salt-affected soil. Knowledge of the genetic diversity and genomics of salt tolerance and identification of genomic regions associated with salt tolerance at the seed germination stage will greatly facilitate breeding salt-tolerant lettuce varieties. In a previous study, four lettuce varieties were tested for their germination ability under salt stress at 0, 50, 100, and 150 mM NaCl, where the varieties Vista and Verte were highly sensitive, and Romaine and Augusta were less sensitive to higher levels of salt stress, demonstrating variability for salt tolerance among the four varieties [14]. In another study, significant variation in seed germination among 27 lettuce varieties was observed under two salt stress conditions (150 and 200 mM NaCl) [12]. Substantial variation in salt tolerance among 178 lettuce cultivars and germplasm accessions from four major horticultural types (butterhead, crisphead, romaine, and leaf) and wild type was reported from a greenhouse study [15]. Reports on molecular markers associated with salinity tolerance in lettuce are limited and to our knowledge, there has been no marker-trait association study on salt tolerance at the seed germination stage in lettuce. An earlier study, using AFLP and EST markers and a recombinant inbred line (RIL) population derived from a bi-parental cross between *L*. *sativa* and *L*. *serriola* identified nine quantitative trait locus (QTLs) including three major QTLs associated with salt-induced changes in root system architecture and ion accumulation at the seedling stage [16]. In a separate study, using an F_2_ population derived from *L*. *sativa* x *L*. *serriola* and 384 SNP markers, four QTLs on chromosome 7 and two QTLs on chromosome 9 for salt tolerance (ion content traits) were identified at seedling stage (35 days old) in lettuce [17]. It should be noted here that for both the above-mentioned studies, seeds were first germinated in non-salt-stress condition followed by salt-stress at the seedling stage [16, 17].

GWAS serves as a model for detecting associations between genotypic and phenotypic values. GWAS has emerged as a powerful tool to resolve complex trait variation at the sequence level using historical and evolutionary recombination events at the population level [18, 19]. GWAS is made possible by the existence of linkage disequilibrium, as it detects and locates QTL based on the strength of correlation between mapped genetic markers and the traits under investigation [20]. GWAS relies on linkage disequilibrium that is initially present in a population, and over several generations, in an unstructured population, only correlations between markers closely linked to QTL remains [20]. However, in the presence of population structure, family structure and cryptic relatedness, marker-trait associations detected by GWAS can be spurious [20–22]. Therefore, to avoid spurious associations, it is important to correct for population structure, family structure and cryptic relatedness in GWAS. Currently, GWAS models are available such as MLM that uses both fixed and random effects and corrects for population structure, family structure and cryptic relatedness [23, 24]. In fact, mixed models are ideal for GWAS, as they can be applied without explicit identification of relatedness within the samples [22]. MLM is a single-locus model that tests one marker at a time, iteratively for every marker in a dataset, while multi-locus models consider the information of all loci simultaneously [25]. When multiple loci contribute to the phenotypic variation of a trait in an additive fashion, single-locus models are not adequate in finding marker-trait associations [26]. To overcome this shortcoming, multi-locus models such as Multiple Locus Mixed Linear Model (MLMM) [27], Fixed and random model Circulating Probability Unification (FarmCPU) [25], and Bayesian-information and Linkage-disequilibrium Iteratively Nested Keyway (BLINK) [28] among others have been developed in recent years.

To our knowledge, there is no report of GWAS on salt tolerance in lettuce. GWAS has been applied to identify marker-trait associations for other traits in lettuce [29–33]. However, GWAS on salt tolerance at the seed germination stage has been reported in other crops such as rice, mungbean, flax and *Brassica napus* [34–37]. Using high density SNPs and 478 accessions, 11 SNP loci associated with salinity susceptibility index of seed germination-related traits under 60 mM NaCl salt stress condition were identified in rice [34]. Using GWAS, SNP loci associated with seed germination percent under 50 mM NaCl salt stress were identified on mungbean chromosomes 7 and 9 [35]. GWAS was performed with 674,074 SNPs and 200 diverse flax accessions, and many SNPs located on all 15 flax chromosomes associated with salt tolerance at the seed germination stage were identified [36]. In another study, GWAS on salt tolerance at the seed germination stage identified 31 salt-stress related QTLs in *B*. *napus* [37].

In an earlier GWAS on bolting in lettuce that included 400 accessions common to the current study, genetic diversity and population structure of the GWAS panel was reported [31]. The objective of the present study was to perform a genome-wide association analysis in lettuce to identify SNP loci significantly associated with salt tolerance at the seed germination stage, which could potentially be used for marker development for breeding improvement of lettuce for salinity tolerance. However, since 45 accessions in the current study were different from the accessions used in the earlier study [31], we also studied population structure and linkage disequilibrium and briefly discuss them in this study.

## Materials and methods

### Plant materials

A set of 445 diverse lettuce accessions (*Lactuca spp*.), mainly consisting of cultivated types with a few wild and primitive types, was selected from the germplasm collection at the USDA-ARS, Salinas, CA for the current study. In an earlier study, a total of 400 accessions common to this diversity panel was used for a genetic diversity and genome-wide association analysis for bolting in lettuce by our group [31]. Based on horticultural types, the 445 accessions in the current study included 117 butterhead, 106 romaine, 100 leaf, 86 crisphead, eight stem, seven wild type (*L*. *serriola*), five primitive, four transitional, three Batavia and one of each of Latin, lobbed, and *L*. *perennis* type (S1 Table).

### Phenotyping and phenotypic data analyses

Phenotypic data were collected through replicated seed germination experiments using petri dishes and filter papers in a germination chamber (Percival, model GR41L; Perry, IA). The experiment involved 150 seeds per accession and was evaluated under two treatment conditions: salt-stress and control. Salt-stress condition was 100 mM NaCl solution prepared in deionized water, while the control condition used deionized water only. A completely randomized experimental design with three replications for both salt-stress and control conditions was employed. Twenty-five seeds for each replication were placed in a 100 mm X 20 mm petri dish containing a single layer filter paper, and then 4.5 mL of NaCl solution and 4.5 mL of deionized water were added, respectively, to the petri dishes with salt-stress and control conditions. A temperature of 21°C and continuous light conditions were maintained in the germination chamber during seed germination. Seed germination was recorded 2 days and 5 days post seeding. A seed was considered germinated when its radicle length reached ≥ 5 mm and cotyledon leaves were open.

GR and SSI were calculated from the seed germination data. GR was expressed as the germination percentage, calculated as GR = N_t_/N_0_ X 100, where N_t_ is the number of seeds germinated on a particular day and N_0_ is the total number of seeds. SSI was calculated following Fischer and Maurer [38] as SSI = (1 − Y_s_/Y_p_)/D, where Y_s_ = mean performance of a genotype under salt stress; Y_p_ = mean performance of the same genotype without salt stress; D (stress intensity) = 1 − (mean Y_s_ of all genotypes/mean Y_p_ of all genotypes).

Statistical analyses included analysis of variance (ANOVA) and Pearson’s correlation coefficient. Using the mean square values from ANOVA, broad-sense heritability (H^2^) on entry-mean basis for GR under salt stress condition and SSI were estimated after Fehr [39] as H^2^ = σ^2^_g_ /(σ^2^_g_ + σ^2^_e_/r), where σ^2^_g_ = genetic variance, σ^2^_e_ = error variance, r = number of replication, and (σ^2^_g_ + σ^2^_e_/r) = phenotypic variance. GR and SSI data were normalized using the rank-based inverse normal transformation [40] for ANOVA and heritability estimates. This transformation method was chosen, because the more commonly used data normalization methods such as log, square root, Box-Cox transformation did not adequately normalize our data set.

### SNP genotyping

Genomic DNA extraction and SNP genotyping were conducted by Data2Bio (Ames, IA) from leaf tissue of 528 lettuce accessions. Genotyping was carried out using a modified tGBS® Genotyping and Sequencing technology with the restriction enzyme BSP1682I [41]. Samples were sequenced using an Illumina HISeq X instrument, and the reads were aligned to the *Lactuca sativa* v8 reference genome (Reyes-Chin-Wo et al. [42]; genome assembly: Lsat_Salinas_v8) after debarcoding and trimming of reads. Data2Bio filtered the SNP sites that meet the tGBS® genotyping criteria resulting in 539,061 SNPs. The initial filtering criteria included: a minimum call rate ≥ 20%, allele number = 2, number of genotypes ≥ 2, minor allele frequency (MAF) ≥ 1%, and heterozygosity rate range = 0%–(2 x Frequency of allele_1_ x Frequency of allele_2_ + 20%). The missing rate of SNPs in each sample was plotted against the fraction of SNPs that were heterozygous and 37 samples were removed due to high missing rate, leaving 491 samples. Due to insufficient seed quantities, only 445 accessions were used in the present study from this set of 491 samples. To retain high-quality SNPs for marker-trait association analyses, SNPs were further filtered with a minimum call rate ≥ 50% and MAF ≥ 5%, resulting in a total of 56,820 SNPs. All downstream analyses were performed using 56,820 SNPs and 445 samples. The missing values were imputed using Beagle version 5.4 software [43] with 3 Burnin iteration and 12 Phasing iteration. This software uses an algorithm that implements haplotype phase interphase and provided a high accuracy imputation with an error rate of < 0.01.

### Analysis of linkage disequilibrium

LD (as measured by r^2^) between pairs of SNPs was computed by TASSEL software version 5.2.87 [44] with 50 sliding windows. LD and LD decay graphs were created using the R program, based on implementation in Remington et al. [45] by plotting LD (r^2^) values against the corresponding genetic distances (bp). Genome-wide LD and LD decay were computed for the set including all accessions (445 accessions = cultivated + wild type + non-cultivated) and for the set with only cultivated accessions (417) from the GWAS panel. Chromosome-wise LD gives associations between alleles on the same chromosome. Therefore, LD and LD decay were also computed individually for each of the nine chromosomes across all (445) accessions. The LD decay distance in base pairs was determined when LD declined to half of the maximum r^2^ value.

### Analysis of population structure

In GWAS, population structure can lead to spurious association if not properly corrected [21]. To address this, we employed principal component analysis (PCA) for detection and correction of population structure using the TASSEL software. PCA is an effective approach for population structure study, and it is computationally tractable when GWAS data set is large [46, 47], such as the 56,820 SNPs in our data set. For visualization of population structure, PC1 and PC2 were plotted using the R package ggplot2 [48]. A total of 424 accessions including the five cultivated horticultural types butterhead, crisphead, leaf, romaine, and stem and the wild type, *L*. *serriola* were used for the PCA. Other types of accessions with limited numbers (1 to 4) were excluded from the analysis.

### Genome-wide association study

Both single-locus and multi-locus models were employed for GWAS using the genotypic data consisting of 56,820 high-quality SNPs and phenotypic data for GR5d_S and SSI_GR5d from the 445 lettuce accessions using the software packages GAPIT version 3 [49] and TASSEL. Phenotypic data was normalized using the rank-based inverse normal transformation, which is widely used for GWAS [40, 50, 51]. The models: General Linear Model (GLM) [46], MLM, MLMM, FarmCPU, and BLINK were implemented using GAPIT. The models GLM and MLM were also implemented using TASSEL. GLM and BLINK use fixed-effect, FarmCPU is a hybrid model that uses both the fixed-effect and the random effect, while MLM and MLMM use a fixed and random effects mixed model. GLM and MLM are single-locus models while FarmCPU, MLMM and BLINK are multi-locus models. To determine how many PCs were needed to correct population structure, three PCs were used in a Bayesian information criterion (BIC)-based model selection procedure implemented in GAPIT. The result indicated that no principal components (PCs) were required to correct population structure in the GWAS models. Accordingly, all GWAS models in GAPIT were run without any PCs. However, the two models in TASSEL were run using the default settings that uses 5 PCs. Manhattan plot was used for visualizing and identifying significant SNPs above the threshold level, while Q-Q plot was used to evaluate model fitness. GAPIT created Manhattan and Q-Q plots were used for the models implemented by this software, while for the models implemented in TASSEL, the Manhattan and Q-Q plots were created from the TASSEL association analysis output files using the R package “qqman” [52]. Bonferroni corrected *P* value threshold (0.05/56820 SNPs; -log_10_(*P*) = 6.06) was used for identifying significant SNPs in the marker-trait association. Further, a significant SNP was considered stable if it was detected by at least two models or software packages or was identified for both the traits, since the two traits are essentially the same.

### Favorable allele effect

To determine which allele of each of the significant SNPs contributed favorably to salt tolerance at the seed germination stage, the average trait value of the two types of homozygous genotypes (homozygous for one or the other allele) at a SNP locus was compared using Kruskal-Wallis test [53]. Heterozygous genotypes were excluded in this calculation to avoid complications arising from any dominance or heterotic effect.

### Identification of putative candidate genes

We used the National Center for Biotechnology Information (NCBI) BLAST to identify putative candidate genes. Considering the presence of highly associated SNPs within the LD decay physical distance in our study and with an objective of detecting the closest genes to the highly associated SNPs, following the recommendations in the article by Alqudah et al. [54], we searched the putative candidate genes within a genomic region spanning 100 Kb upstream and downstream of each significant SNP locus. Both the v8 and v11 (Reyes-Chin-Wo et al. [42]; genome assembly: Last_Salinas_v11) versions of the lettuce reference genomes were used for the candidate gene search.

## Results

### Variability and correlation

Mean, standard error (S.E.), and range for the germination related traits measured under control (water) and salt stress conditions are given in Table 1. Mean GR in water, 2 days post seeding (GR2d_W) and 5 days post seeding (GR5d_W) were 97% and 99%, respectively, while the ranges were 21% to 100% and 84% to 100%, respectively. Mean GR under salt stress condition, 2 days post seeding (GR2d_S) and 5 days post seeding (GR5d_S) were 62% and 77%, respectively, while the range was 0% to 100% for both traits. Mean and ranges for the SSI, 2 days post seeding (SSI_GR2d) were 1.02 and -0.18 to 2.81 while those for the SSI, 5 days post seeding (SSI_GR5d) were 1.0 and -0.26 to 4.38, respectively. For SSI, the lower the value the better the salinity tolerance for that genotype, since SSI is a susceptibility index. The SSI has been used to evaluate genotype performance under stress and non-stress conditions by several other studies [34, 55–57].

**Table 1:** Mean, standard error (S.E.), and range for seed germination related traits of 445 lettuce accessions evaluated in germination chamber under control (water) and salt stress (100 mM NaCl) conditions.

Trait	Mean ± S.E.	Range
Germination rate in water, 2 days post seeding (GR2d_W)	96.95 ± 0.37	21.33–100.00
Germination rate in water, 5 days post seeding (GR5d_W)	99.29 ± 0.08	84.00–100.00
Germination rate in salt, 2 days post seeding (GR2d_S)	62.43 ± 1.57	0.00–100.00
Germination rate in salt, 5 days post seeding (GR5d_S)	76.63 ± 1.38	0.00–100.00
Salinity susceptibility index, 2 days post seeding (SSI_GR2d)	1.02 ± 0.02	-0.18–2.81
Salinity susceptibility index, 5 days post seeding (SSI_GR5d)	1.00 ± 0.06	-0.26–4.38

**Fig 1:**
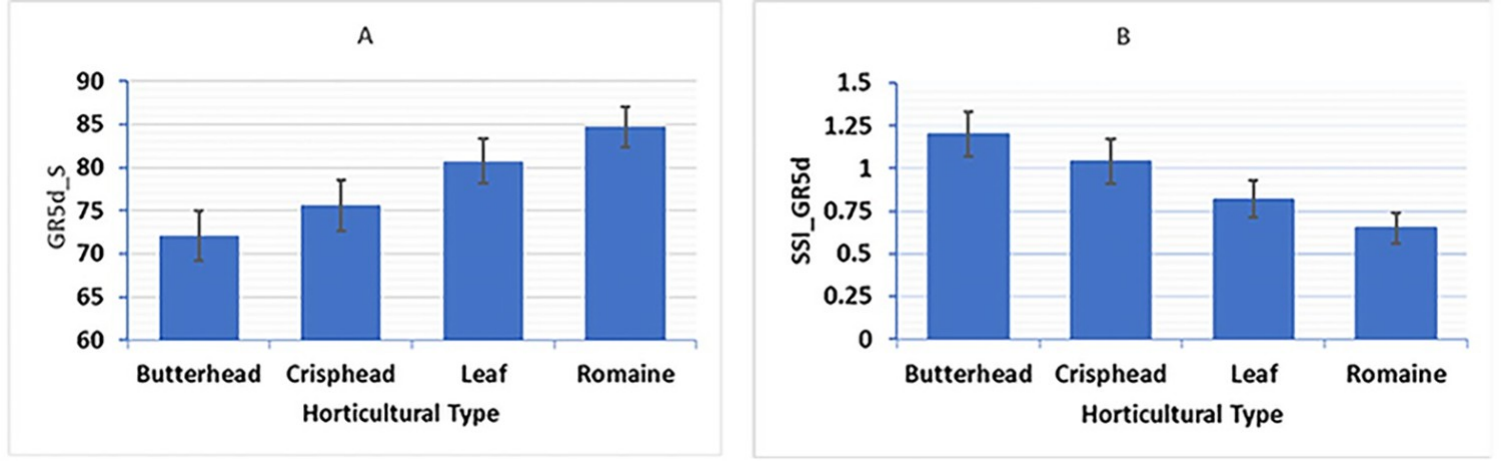
Bar graphs of the means for the two traits for the four major horticultural types of lettuce. A) Mean germination rate under salt stress, 5 days post seeding (GR5d_S), B) Mean salinity susceptibility index, 5 days post seeding (SSI_GR5d). Error bars indicate the standard errors of the means.

Mean and standard errors for GR5d_S and SSI_GR5d of the four major horticultural types (butterhead, crisphead, leaf and romaine) are presented by bar graphs (Fig 1). Under salt stress condition, the romaine type had the highest mean GR5d_S of 85%, while the butterhead type had the lowest mean GR5d_S of 72%. The leaf and the crisphead types had mean GR5d_S of 81% and 76%, respectively. Similarly, the mean SSI_GR5d was lowest (0.65) for the romaine type and highest (1.2) for the butterhead type, indicating the highest salinity tolerance at the seed germination stage for the romaine type. ANOVA indicated significant differences (*P* < 0.001) among the 445 lettuce accessions for GR2d_S, GR5d_S, SSI_GR2d, and SSI_GR5d (S2 Table). Genetic variance ranged from 0.695 for GR5d_S to 0.799 for SSI_GR2d. Broad-sense heritabilities were 0.92 and 0.93 for the traits SSI_GR5d and SSI_GR2d, respectively, and 0.95 for both GR5d_S and GR2d_S (S2 Table).

Correlation coefficients among the seed germination-related traits evaluated under control and salinity stress conditions are given in Table 2. All 15 correlations among the traits were significant at *P* < 0.01. The coefficients/values were higher for some correlations than for others. For instance, the correlation coefficient between GR2d_W and GR5d_W was 0.705, while it was 0.869 between GR2d_S and GR5d_S. Correlation coefficients between GR in water and GR under salt stress were lower (r = 0.195 to r = 0.319). This discrepancy can be attributed to the fact that most accessions germinated well under control (water) condition, while germination rates varied highly under salt stress conditions due to the differences in salt tolerance among the accessions. This can be observed from the lower mean value and wider range for germination rates under salt stress than under control condition (Table 1). The correlation coefficient between GR5d_S and SSI_GR5d was r = -0.999 (R^2^ = 0.998), while that of between GR2d_S and SSI_GR2d was r = -0.998 (R^2^ = 0.996). These later two correlation coefficients were high but negative since salinity tolerance implies higher germination rate but lower salinity susceptibility index.

**Table 2:** Correlation coefficients among several seed germination related traits evaluated under control (water) and salt stress (100 mM NaCl) conditions in lettuce.

	GR5d_W	GR2d_S	GR5d_S	SSI_GR2d	SSI_GR5d
GR2d_W	0.705	0.286	0.319	-0.264	-0.311
GR5d_W		0.195	0.218	-0.174	-0.193
GR2d_S			0.869	-0.998	-0.866
GR5d_S				-0.871	-0.999
SSI_GR2d					0.870

All correlations are significant at *P* < 0.01.

GR2d_W and GR5d_W are germination rates in water, 2 days and 5 days post seeding, respectively.

GR2d_W and GR5d_S are germination rates under salt stress, 2 days and 5 days post seeding, respectively.

SSI_GR2d and SSI_GR5d are salinity susceptibility index, 2 days and 5 days post seeding, respectively.

### Population structure

**Fig 2:**
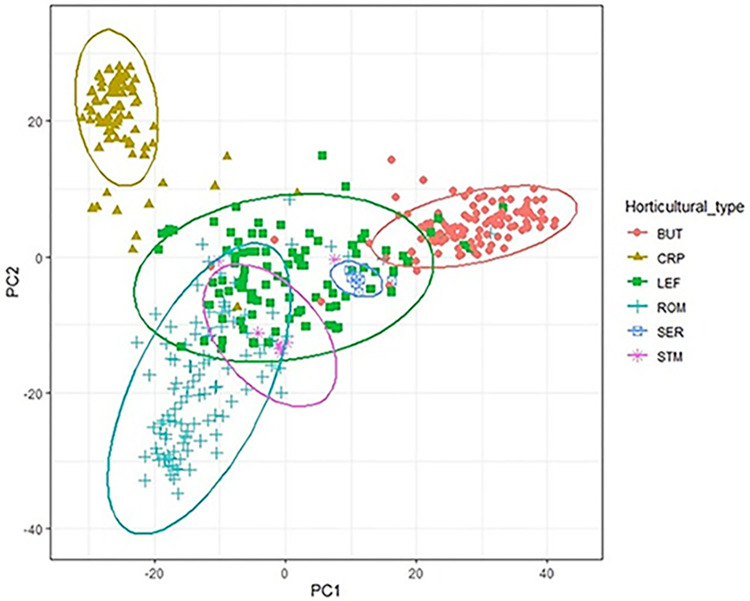
PCA plot of the five horticultural types and the wild-type lettuce in the GWAS panel. Butterhead (red, round), crisphead (green, triangle), leaf (green, square), romaine (blue, plus), *L*. *serriola* (blue, square cross), stem (blue, star). Ellipses indicate 95% confidence level for a bi-variate t-distribution.

We employed PCA to study population structure using 424 accessions including the five cultivated horticultural types and the wild type, *L*. *serriola* lettuce (Fig 2). The ellipses around the clusters in the scatterplot represented a 95% confidence level for a bi-variate t-distribution. PC1 and PC2 accounted for 11.1% and 5.4% of the total variation, respectively, collectively accounting for 16.5% of the total variation. The accessions clustered predominantly based on their horticultural types. The cluster of the leaf type accessions showed complete overlap with the *L*. *serriola* type cluster and nearly complete overlap with the stem type. The leaf type also exhibited considerable overlap with the romaine type and some overlap with the butterhead type. Although the number of accessions from *L*. *serriola* and stem types were smaller compared to the other four horticultural types in this study, the clustering indicated a genetic closeness of the leaf type to the *L*. *serriola* and the stem types. There was no overlap between the butterhead and romaine types, however, some genetic closeness was apparent among the leaf, romaine and butterhead types (Fig 2). In contrast, the crisphead type did not overlap with any other types, indicating that it was relatively genetically distant from the other horticultural types of lettuce. It has been reported that romaine lettuce developed from the wild lettuce while leaf, butterhead, and Batavia-crisphead types originated from the romaine type and the stem type was later derived from the leaf type [6]. Even later, the iceberg type of crisphead lettuce was developed from the Batavia type in the U.S. in the 1940s [8]. Overall, the PCA plot indicated presence of population stratification among the 424 lettuce accessions in our GWAS panel.

### Genome-wide SNP distribution

**Fig 3:**
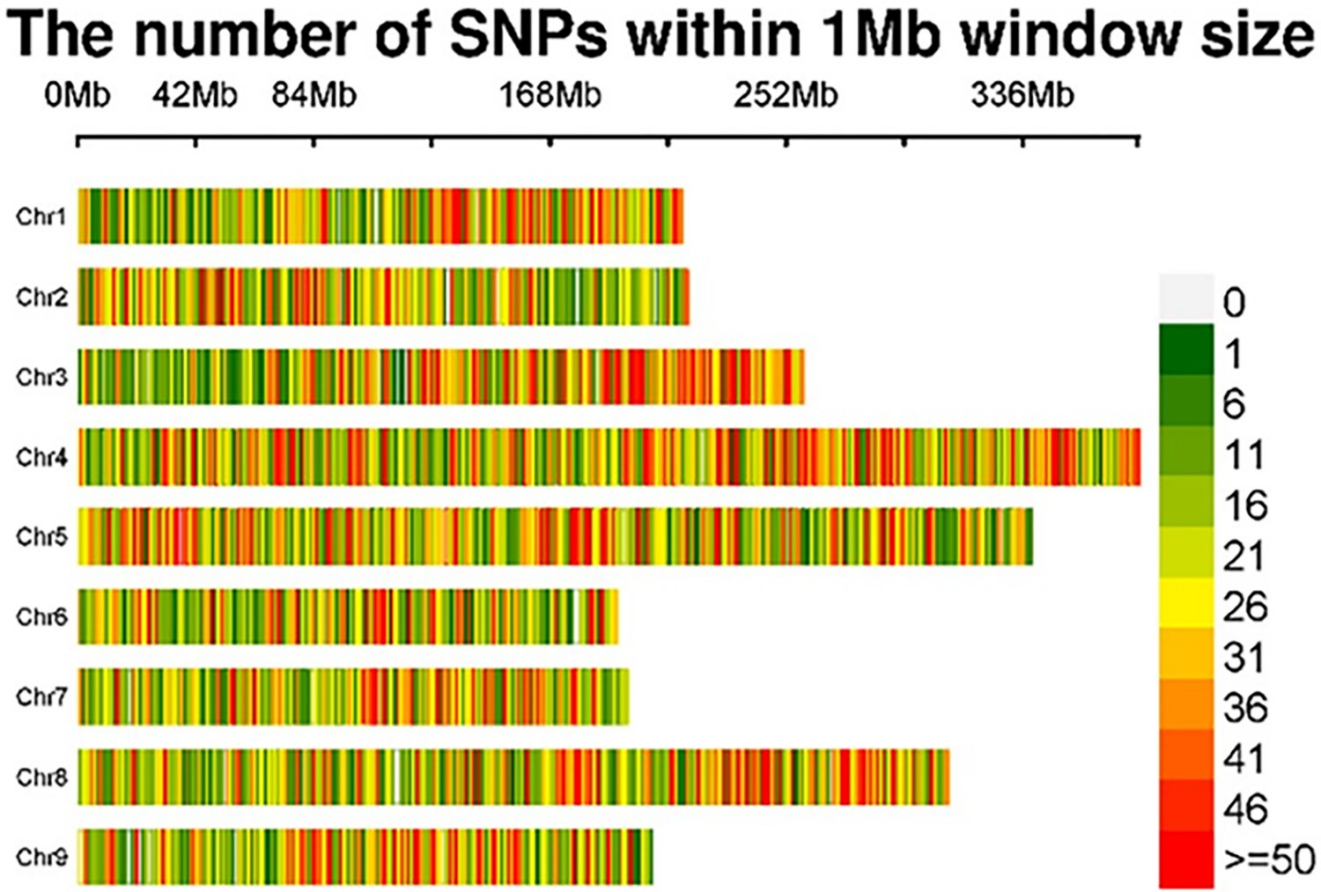
SNP density plot on 9 lettuce chromosomes showing number of SNPs within 1 Mb window size. The x-axis represents chromosome length in Mb. Plotted by https://www.bioinformatics.com.cn/en, a free online platform for data analysis and visualization.

The total number of genome-wide SNPs for this study was 56,820 high-quality SNPs with a filtering criteria of minimum call rate ≥ 50% and MAF ≥ 5%. These SNPs were identified using lettuce reference genome version v8. The size of this reference genome is 2.37 Gb. Thus, the average genome-wide SNP coverage was 24 SNPs/Mb. Chromosome-wise distributions of the SNPs on nine lettuce chromosomes are given in Table 3 and Fig 3. Chromosome size varied from 193.11 Mb (chromosome 6) to 399.64 Mb (chromosome 5). The number of SNPs on individual chromosomes ranged from 4,189 on chromosome 6 to 10,112 on chromosome 4. SNP density on individual chromosomes ranged from 21.43/Mb on chromosome 5 to 26.78/Mb on chromosome 4.

**Table 3:** Chromosome-wise SNP distribution on nine lettuce chromosomes in reference genome v8.

Chromosome	Chromosome size (bp)	Number of SNPs	SNP density per Mb
1	214,790,941	5,224	24.32
2	217,164,977	4,958	22.83
3	257,910,700	6,518	25.27
4	377,529,803	10,112	26.78
5	399,640,866	8,565	21.43
6	193,110,136	4,189	21.69
7	195,685,357	4,510	23.05
8	309,698,552	7,759	25.05
9	204,289,203	4,985	24.40

### Linkage disequilibrium and LD decay

**Fig 4:**
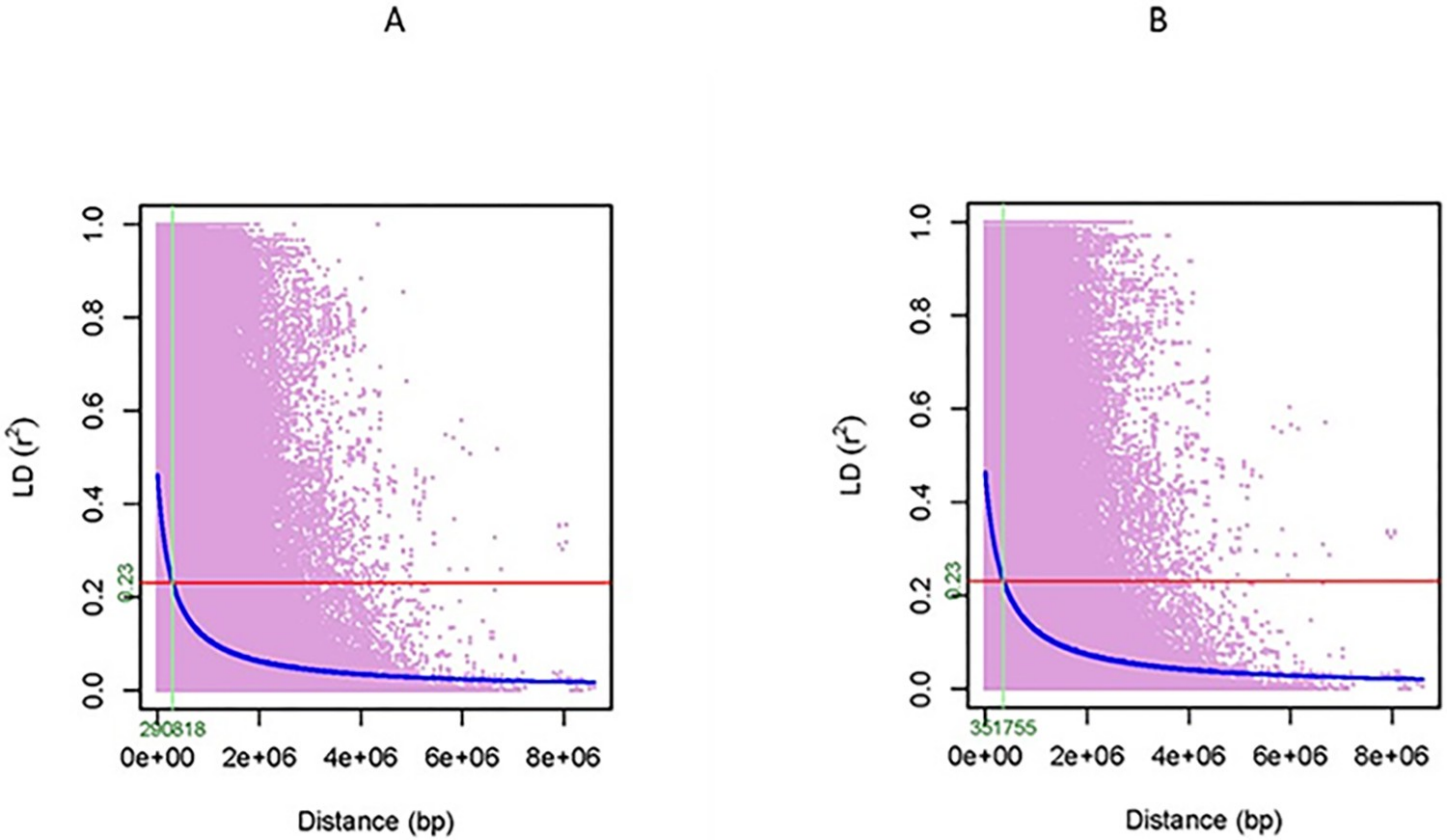
Linkage disequilibrium (LD) and LD decay graphs, based on the whole genome of lettuce. A) For the 445 accessions including cultivated, wild, and non-cultivated types, and B) for the 417 cultivated accessions of lettuce. LD decayed to half of its maximum at 290818 bp for the 445 accessions, and at 351755 bp for the 417 accessions.

A total of 342,637 pairwise LD values were found to be significant (*P* ≤0.05) based on the genome-wide LD for the set of 445 accessions with an average LD of 0.407, while 327,466 pairwise LD values were significant (*P* ≤0.05) for the set of 417 cultivated accessions with an average LD of 0.435 (Table 4). The genome-wide LD for the set of 445 accessions decayed to half of its maximum value at a distance of 290.8 Kb, while the genome-wide LD for the set of 417 cultivated accessions decayed to half at 351.8 Kb (Fig 4). This difference can be attributed to the presence of seven wild type (*L*. *serriola*) and 21 non-cultivated lettuce in the set of 445 accessions. The pairwise LD comparison for individual chromosomes showed that chromosome 4 had the maximum number of 72,572 significant (*P* ≤0.05) LD values, with an average LD of 0.423, whereas chromosome 6 had the minimum number of 11,816 significant (*P* ≤0.05) LD values with an average LD of 0.372 (Table 4). Chromosome 6 had the fastest LD decay to half of its maximum at 89.3 Kb, while chromosome 4 had the slowest LD decay at 403.7 Kb (S1 Fig).

**Table 4:** Chromosome-wise (445 accessions) and genome-wide linkage disequilibrium (LD) and LD decay to half of its maximum in the lettuce GWAS panel.

Chromosome-wise/genome-wide	No. of SNP pairs in LD with P ≤ 40.05	Average LD (r2) of SNP pairs with P ≤ 0.05	LD decay distance (bp)
1	44,051	0.346	261,664
2	29,440	0.377	199,542
3	37,726	0.460	349,541
4	72,572	0.423	403,689
5	48,268	0.340	259,715
6	11,816	0.372	89,382
7	23,784	0.376	232,815
8	47,328	0.430	332,532
9	27,163	0.447	328,898
Genome-wide (445 taxa)^a^	342,637	0.407	290,818
Genome-wide (417 taxa)^b^	327,466	0.435	351,755

a, Genome-wide LD and LD decay computed with all 445 accessions in the study.

b, Genome-wide LD and LD decay computed with only cultivated type accessions (417).

### Genome-wide association study

Using the 56,820 high-quality SNPs, GWAS was performed on the two traits (GR5d_S and SSI_GR5d) related to salt tolerance at the seed germination stage using five GWAS models and two software packages: GAPIT (GLM, MLM, MLMM, FarmCPU, BLINK) and TASSEL (GLM, MLM). Phenotypic data was normalized using the rank-based inverse normal transformation. Distribution of the residuals from GWAS using MLM in TASSEL are presented in S2 and S3 Figs, respectively, for the two traits GR5d_S and SSI_GR5d. Although the lettuce accessions used in the present study indicated presence of population structure (Fig 2), the BIC-based model selection procedure in GAPIT indicated that no PCs were required for correction of population structure in the GWAS models for both traits (S3 Table). However, the BIC values for 0, 1, 2 and 3 PCs were not very different from each other, therefore, we conducted GWAS using GAPIT with 0, 1, 2, and 3 PCs in the GWAS models for comparison of the results (S4 and S5 Tables). Comparison of the results for both traits indicated that identified SNPs were more consistent across models when no PCs were provided in the GWAS models. Therefore, we used the results from the analyses using no PCs in the GWAS models in GAPIT (S4 and S5 Tables). We also conducted GWAS using the GLM and MLM models in TASSEL with the default parameters that uses 5 PCs in the GWAS model, and the results are presented in S6 and S7 Tables, respectively, for the traits GR5d_S and SSI_GR5d.

**Fig 5:**
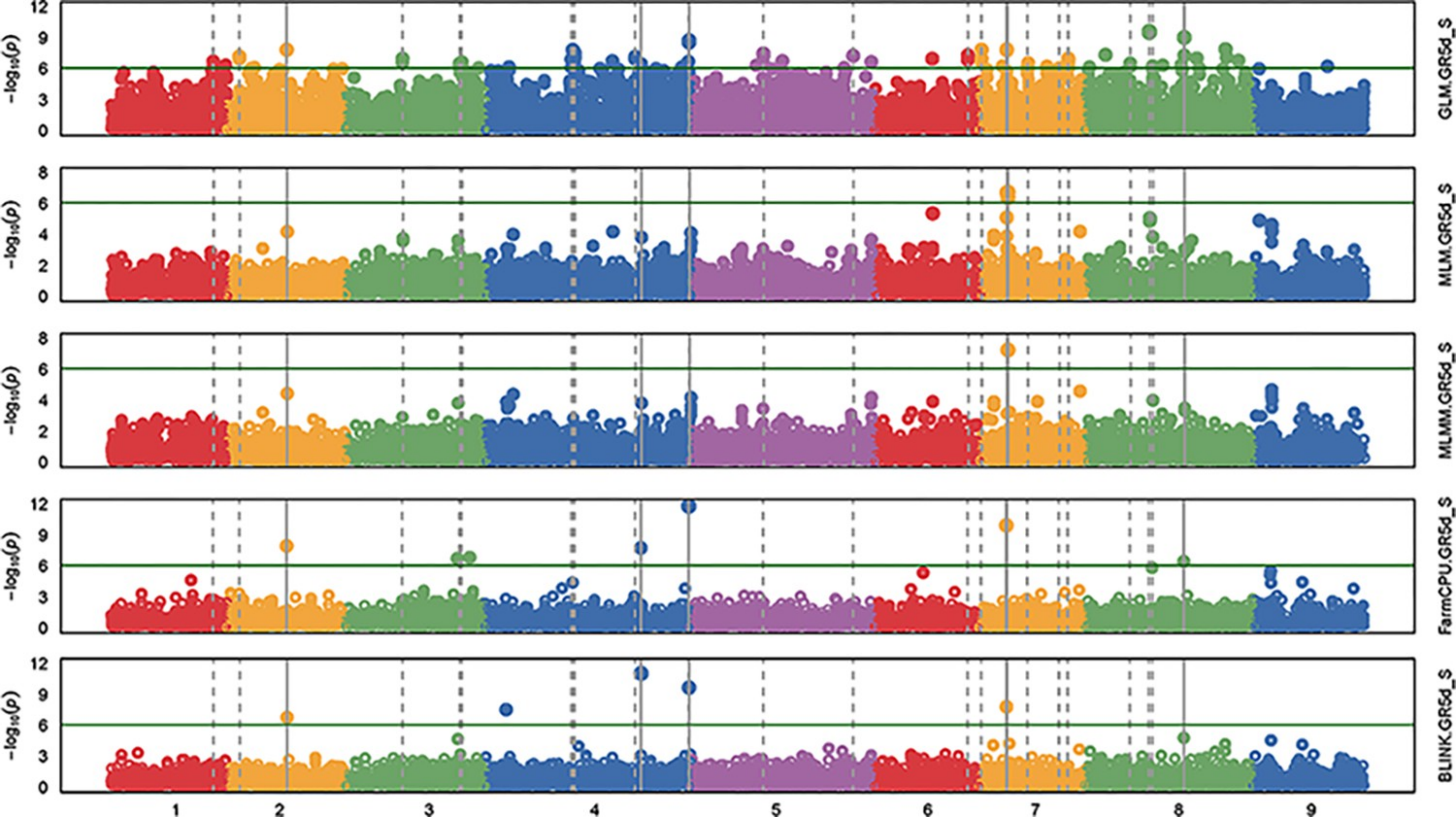
Manhattan plots of the GWAS results for the trait germination under salt stress, 5 days post seeding (GR5d_S) using GAPIT. The genome-wide association study (GWAS) results from the GAPIT software package involved 445 lettuce accessions and 56,820 SNPs. The five plots from top to bottom are based on the GWAS models: general linear model (GLM), mixed linear model (MLM), Multiple Locus Mixed Linear Model (MLMM), Fixed and random model Circulating Probability Unification (FarmCPU), and Bayesian-information and Linkage-disequilibrium Iteratively Nested Keyway (BLINK). The X-axis shows the genomic positions of the SNPs and the Y-axis shows the negative log base 10 of the *P*-values. Each of the nine lettuce chromosomes are represented with different colors. The horizontal line represents the Bonferroni correction threshold for significant marker-trait association.

**Fig 6:**
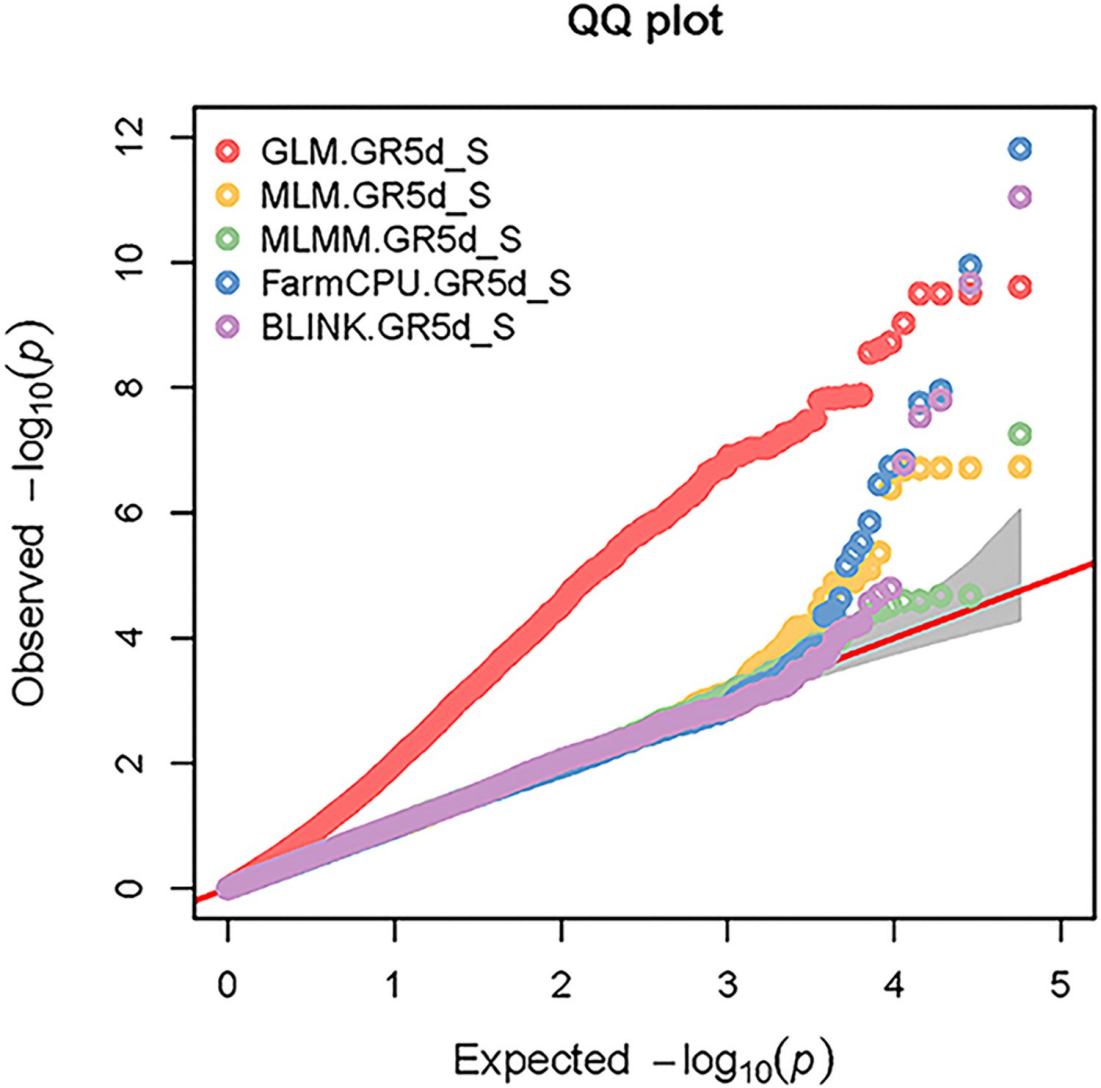
**Quantile-quantile (Q-Q) plots of the GWAS results for the trait germination under salt stress, 5 days post seeding (GR5d_S) using GAPIT.** The genome-wide association study (GWAS) results from the GAPIT software package involved 445 lettuce accessions and 56,820 SNPs. The Q-Q plots are for the five GWAS models: general linear model (GLM), mixed linear model (MLM), Multiple Locus Mixed Linear Model (MLMM), Fixed and random model Circulating Probability Unification (FarmCPU), and Bayesian-information and Linkage-disequilibrium Iteratively Nested Keyway (BLINK). The Y-axis and X-axis represent, respectively, the observed and the expected, negative log base 10 of the *P*-values. The dotted lines represent the 95% confidence interval for the Q-Q plot under the null hypothesis of no association between the SNP and the trait.

**Fig 7:**
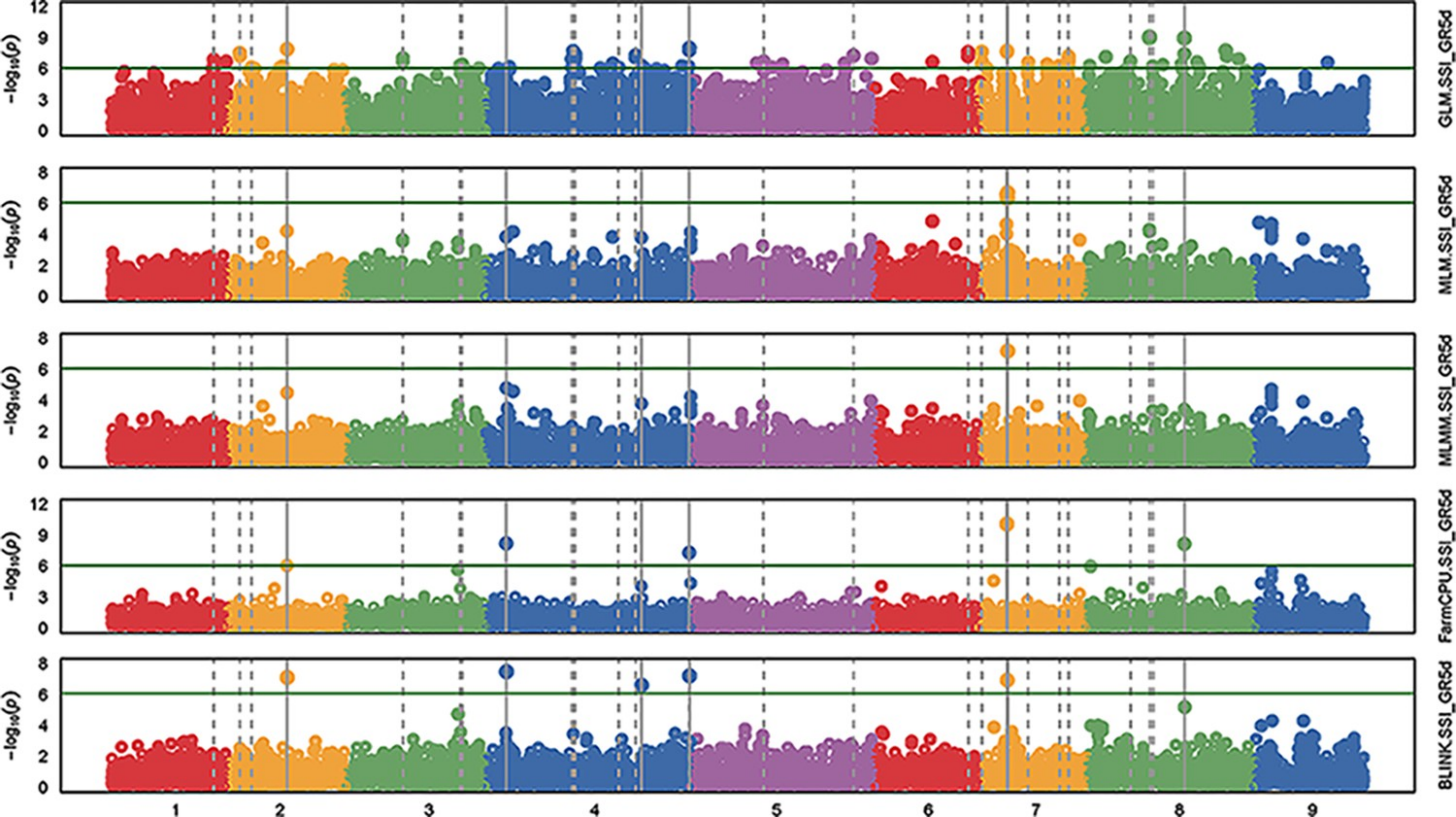
**Manhattan plots of the GWAS results for the trait salinity susceptibility index, 5 days post seeding (SSI_GR5d) using GAPIT.** The genome-wide association study (GWAS) results from the GAPIT software package involved 445 lettuce accessions and 56,820 SNPs. The five plots from top to bottom are based on the GWAS models: general linear model (GLM), mixed linear model (MLM), Multiple Locus Mixed Linear Model (MLMM), Fixed and random model Circulating Probability Unification (FarmCPU), and Bayesian-information and Linkage-disequilibrium Iteratively Nested Keyway (BLINK). The X-axis shows the genomic positions of the SNPs and the Y-axis shows the negative log base 10 of the *P*-values. Each of the nine lettuce chromosomes are represented with different colors. The horizontal line represents the Bonferroni correction threshold for significant marker-trait association.

**Fig 8:**
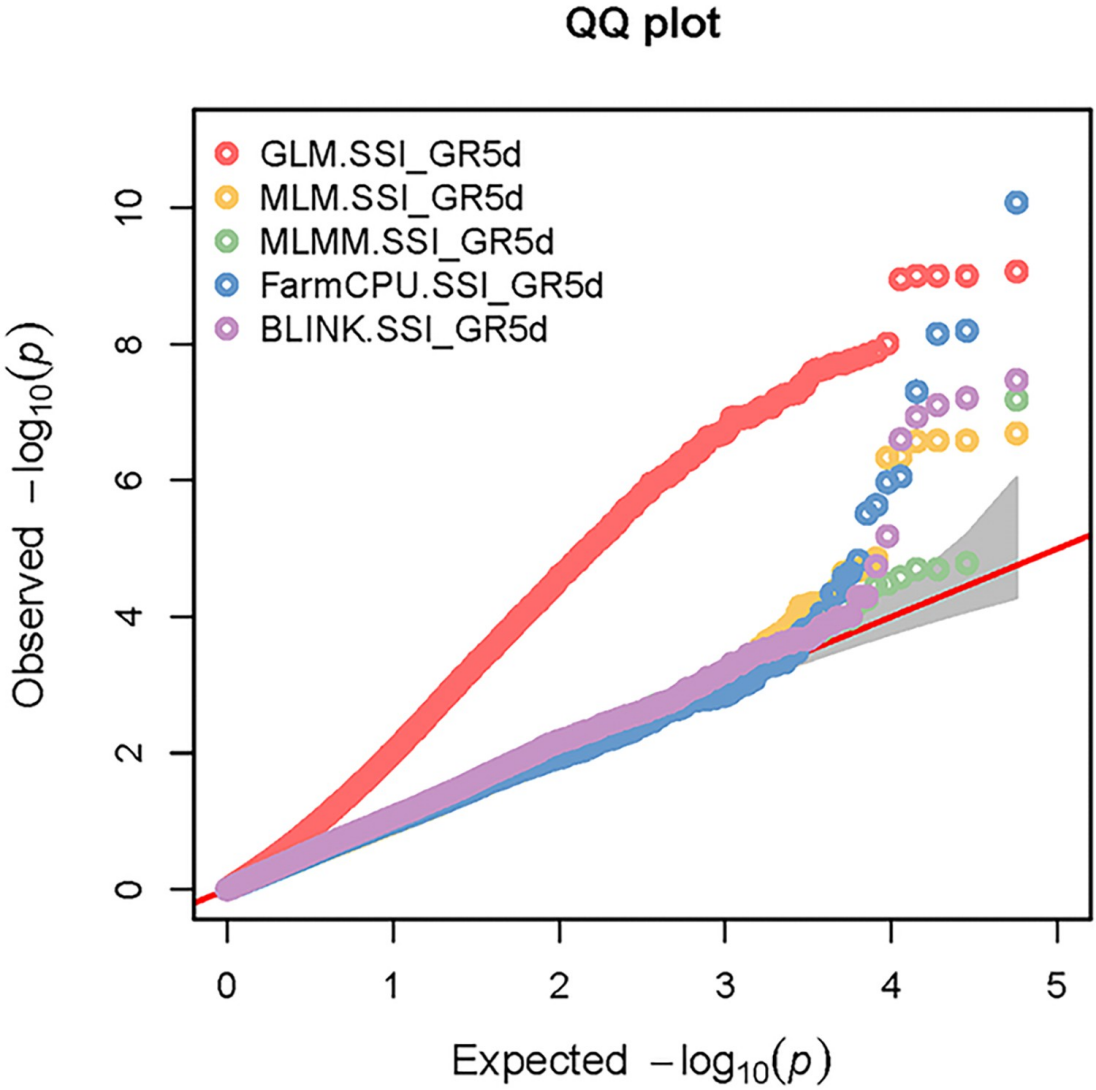
**Quantile-quantile (Q-Q) plots of the GWAS results for the trait salinity susceptibility index, 5 days post seeding (SSI_GR5d) using GAPIT.** The genome-wide association study (GWAS) results from the GAPIT software package involved 445 lettuce accessions and 56,820 SNPs. The Q-Q plots are for the five GWAS models: general linear model (GLM), mixed linear model (MLM), Multiple Locus Mixed Linear Model (MLMM), Fixed and random model Circulating Probability Unification (FarmCPU), and Bayesian-information and Linkage-disequilibrium Iteratively Nested Keyway (BLINK). The Y-axis and X-axis represent, respectively, the observed and the expected, negative log base 10 of the *P*-values. The dotted lines represent the 95% confidence interval for the Q-Q plot under the null hypothesis of no association between the SNP and the trait.

**Fig 9:**
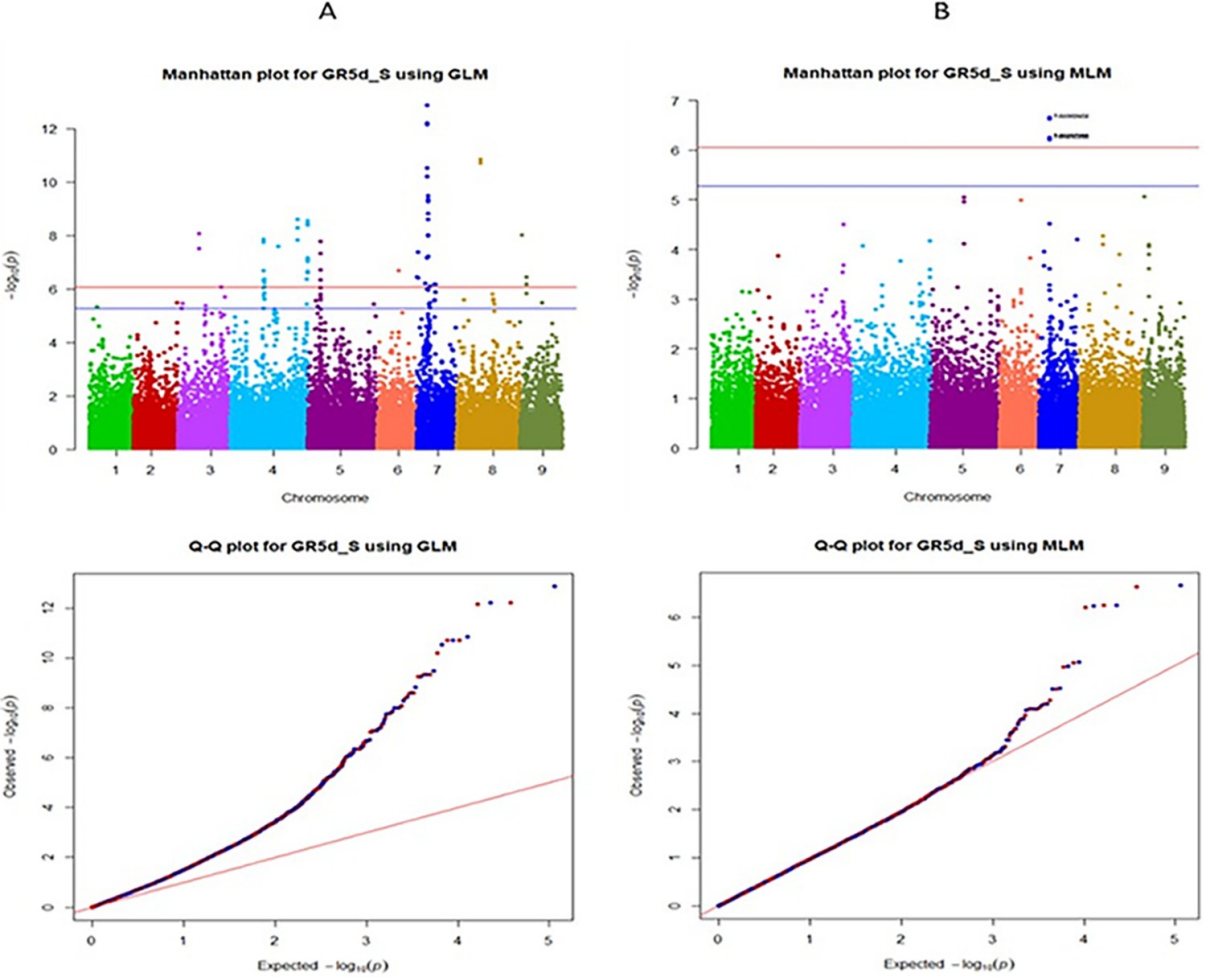
Manhattan and quantile-quantile (Q-Q) plots of the GWAS results for the trait germination under salt stress, 5 days post seeding (GR5d_S) using TASSEL. The genome-wide association study (GWAS) results from the TASSEL software package involved 445 lettuce accessions and 56,820 SNPs. Panel A) Based on the general linear model (GLM), Panel B) Based on the mixed linear model (MLM). The upper and the lower horizontal lines in the Manhattan plots represent the Bonferroni and the Benjamini-Hochberg correction thresholds for significant marker-trait associations, respectively. For the Q-Q plots, the Y-axis and X-axis represent, respectively, the observed and the expected, negative log base 10 of the *P*-values.

**Fig 10:**
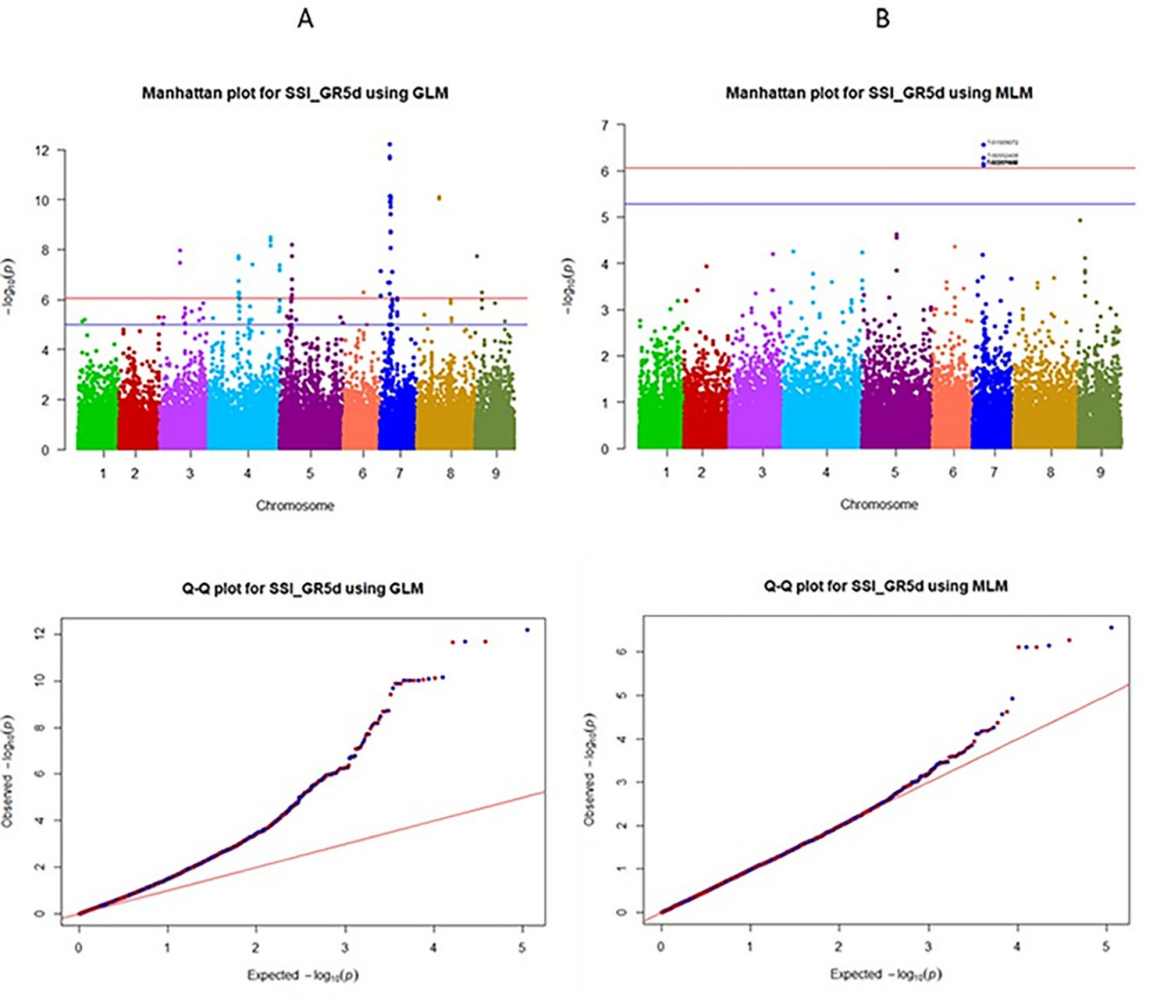
Manhattan and quantile-quantile (Q-Q) plots of the GWAS results for the trait salinity susceptibility index, 5 days post seeding (SSI_GR5d) using TASSEL. The genome-wide association study (GWAS) results from the TASSEL software package involved 445 lettuce accessions and 56,820 SNPs. Panel A) Based on the general linear model (GLM), Panel B) Based on the mixed linear model (MLM). The upper and the lower horizontal lines in the Manhattan plots represent the Bonferroni and the Benjamini-Hochberg correction thresholds for significant marker-trait associations, respectively. For the Q-Q plots, the Y-axis and X-axis represent, respectively, the observed and the expected, negative log base 10 of the *P*-values.

The Manhattan plots and the Q-Q plots from GWAS for all five models using GAPIT are given respectively, in the Figs 5 and 6 for the trait GR5d_S and in the Figs 7 and 8 for the trait SSI_GR5d, while the Manhattan plots and the Q-Q plots for the models GLM and MLM using TASSEL are given in the Figs 9 and 10, respectively, for the traits GR5d_S and SSI_GR5d. For the trait GR5d_S, when GAPIT was used, GLM identified 122 significant SNPs spanning over all nine lettuce chromosomes, MLM identified six significant SNPs on chromosome 7, MLMM identified one SNP on chromosome 7, FarmCPU identified a total of seven significant SNPs on chromosomes 2, 3, 4, 7, and 8, while BLINK identified one SNP on chromosome 2, three SNPs on chromosome 4, and one SNP on chromosome 7 (S4 Table, Fig 5). For GR5d_S, when TASSEL was used, GLM identified 94 significant SNPs on chromosomes 3 through 9, while MLM identified six SNPs on chromosome 7 (S6 Table, Fig 9). It should be noted here that although no PCs were used for MLM in GAPIT and 5 PCs were used for MLM in TASSEL, the same six significant SNPs were identified by both software packages (S4 and S6 Tables). For the trait SSI_GR5d, when GAPIT was used, GLM identified 134 significant SNPs spanning over all nine lettuce chromosomes, MLM identified six significant SNPs on chromosome 7, MLMM identified one SNP on chromosome 7, FarmCPU identified a total of five significant SNPs on chromosomes 2, 4, 7, and 8, while BLINK identified the same four SNPs as for the trait GR5d_S, on chromosomes 2 and 4 and a different SNP on chromosome 7 (S5 Table, Fig 7). For SSI_GR5d, when using TASSEL, GLM identified 72 SNPs on chromosomes 3 through chromosome 9, while MLM identified the same six SNPs on chromosome 7 as for GR5d_S (S7 Table, Fig 10). For GLM, Q-Q plots from both GAPIT and TASSEL, for both the traits showed that the line for the observed *P*-values deviated strongly from the line for the expected *P*-values (the diagonal line), indicating that the confounding effects of population structure, family structure and cryptic relatedness were not adequately corrected by the GLM (Figs 6, 8–10). Thus, although the GLM identified over 100 significant SNPs for both the traits, many of these SNPs might be spurious. On the other hand, the Q-Q plots for the rest of the models for both the traits showed a substantial reduction of the deviation of the observed *P*-values, with only minor deviations towards the tail, suggesting that the models MLM, MLMM, FarmCPU and BLINK effectively corrected for the confounding effects and well-suited to our dataset. It should be noted here that the BIC-based model selection results suggested that no PC input was required for correcting population structure in the GWAS models in GAPIT. Thus, although the PCA in our study detected population structure in our GWAS panel, the population structure might not be strong and the GWAS models were able to effectively remove the confounding effects through the kinship matrix. Therefore, we identified significant SNPs based on the results from the four models MLM, MLMM, FarmCPU and BLINK. SNP significance was based on the Bonferroni correction threshold at α = 0.05, -log_10_(*P*) = 6.06. A significant SNP was considered stable if it was detected by at least two models or software packages or it was identified for both the traits, since the two traits were essentially the same. Based on this criteria, 10 stable SNPs were identified for both the traits (Table 5).

**Table 5:** List of the ten stable significant SNPs associated with the two traits GR5d_S and SSI_GR5d related to salt tolerance at the seed germination stage in lettuce identified in the present study.

SNP^a^	Chr	GR5d_S			SSI_GR5d		
		Model/ Software	-log_10_(*P*)^b^	PVE (%)^c^	Model/ Software	-log_10_(*P*)^b^	PVE (%)^c^
*Ls*v8Lg2-109644094	2	2	6.78	4.28	2	7.11	4.48
*Ls*v8Lg4-38095762	4	2	7.56	3.64	2	7.47	3.90
*Ls*v8Lg4-286540234	4	2	11.05	4.34	2	6.61	4.22
*Ls*v8Lg4-374691029	4	1, 2	9.67–11.82	1.86–8.05	1, 2	7.21–7.31	1.73–11.40
*Ls*v8Lg7-50257083	7	1, 2, 3, 4	6.25–9.95	0.11–6.70	3, 4	6.12–6.59	3.04–6.60
*Ls*v8Lg7-50257096	7	3, 4	6.25–6.72	1.58–6.70	1, 2, 3, 4	6.12–10.08	0.15–6.60
*Ls*v8Lg7-50257104	7	3	6.24	6.70	3	6.12	6.60
*Ls*v8Lg7-50552408	7	3	6.64	7.10	3	6.28	6.80
*Ls*v8Lg7-51509072	7	3, 5	6.66–7.25	5.94–7.20	3, 5	6.57–7.19	5.75–7.10
*Ls*v8Lg7-51616430	7	3, 4	6.21–6.39	1.18–6.70	3, 4	6.16–6.35	1.23–6.60

Model/Software: 1 = FarmCPU, 2 = BLINK, 3 = MLM-TASSEL, 4 = MLM-GAPIT, 5 = MLMM.

^b^, -log10(*P*) of marker-trait association

^c^, PVE = phenotypic variation explained

-log_10_(*P*) for Bonferroni correction threshold = 6.06

Based on the SNP positions, we designated the 10 stable SNPs as *Ls*v8Lg2-109644094, *Ls*v8Lg4-38095762, *Ls*v8Lg4-286540234, *Ls*v8Lg4-374691029, *Ls*v8Lg7-50257083, *Ls*v8Lg7-50257096, *Ls*v8Lg7-50257104, *Ls*v8Lg7-50552408, *Ls*v8Lg7-51509072, and *Ls*v8Lg7-51616430, where *Ls* = *Lactuca sativa*, v8 = version 8 of the reference genome, Lg = chromosome, followed by chromosome number and SNP location. Although both BLINK and FarmCPU identified the four SNPs, *Ls*v8Lg2-109644094, *Ls*v8Lg4-38095762, *Ls*v8Lg4-286540234, and *Ls*v8Lg4-374691029 as significant SNPs, the percent phenotypic variation explained (PVE) by these SNPs in FarmCPU were of magnitude zero to very small, except for *Ls*v8Lg4-374691029, where the PVE were 8% and 11% for the traits GR5d_S and SSI_GR5d, respectively (S4 and S5 Tables). Thus, a SNP with a very small PVE% was not included in deciding the stable SNPs, despite its significance. The six significant SNPs identified by the single-locus model MLM were located within ~ 1.36 Mb on chromosome 7. However, each of the three multi-locus models MLMM, FarmCPU and BLINK identified only one of the six SNPs. The MLMM identified the SNP *Ls*v8Lg7-51509072 for both the traits, while FarmCPU and BLINK both identified the SNP *Ls*v8Lg7-50257083 for the trait GR5d_S and the SNP *Ls*v8Lg7-50257096 for the trait SSI_GR5d. Thus, most likely these six SNPs indicated the same QTL. Considering the six SNPs on chromosome 7 indicating the same QTL, the 10 stable SNPs were associated with five QTLs in the present study (Table 6). Estimates of the PVE% by different models and software packages varied. For example, the average PVE% for the two traits for the SNP *Ls*v8Lg4-374691029 was 10% and 2%, reported respectively, by FarmCPU and BLINK. For the MLM model, TASSEL consistently reported a 6% to 7% average PVE for each of the six SNPs on chromosome 7, while GAPIT reported a 0% to 3% PVE for these SNPs (S4–S7 Tables). Since the PVE% estimated by the BLINK and the MLMM models were consistent for the two traits, we reported the average PVE% of the two traits for each of the five QTLs estimated by these two models (Table 6). The average PVE ranged from 2% for the QTL qSTgs4.3 to 6% for the QTL qSTgs7.1, indicating the quantitative nature of these two salt tolerance traits GR5d_S and SSI_GR5d.

**Table 6:** List of the five QTLs associated with the two traits GR5d_S and SSI_GR5d related to salt tolerance at the seed germination stage in lettuce identified in the present study.

QTL^a^	Chromosome	SNP	PVE (%)
qSTgs2.1	2	*Ls*v8Lg2-109644094	4.38
qSTgs4.1	4	*Ls*v8Lg4-38095762	3.77
qSTgs4.2	4	*Ls*v8Lg4-286540234	4.28
qSTgs4.3	4	*Ls*v8Lg4-374691029	1.80
qSTgs7.1	7	Lead SNP*—Ls*v8Lg7-50257083 End SPN—*Ls*v8Lg7-51616430	5.85

^a^, QTL designation: q = QTL, ST = salt tolerance, gs = germination stage, gs is followed by chromosome number, followed by a period and number of QTLs identified on that chromosome in the present study.

### Mapping SNPs from lettuce reference genome version v8 to reference genome version v11

Variant calls for genotyping for the current project were performed using the latest lettuce reference genome version, v8, available at that time. However, the most recent lettuce reference genome version, v11, was recently submitted to NCBI while we were preparing this manuscript. Total genome size of the reference genomes v8 and v11 are 2.4 Gb and 2.6 Gb, respectively. The 10 significant and stable SNPs identified in the reference genome v8 in the present study were mapped to reference genome v11 (Table 7) and were confirmed by alignment of 2 Kb (±1 Kb) sequence length flanking each SNP site in v8 with the corresponding SNP site in v11. Three of the six SNP loci 50257083, 50257096 and 50257104 on chromosome 7 were within 21 base pairs in the reference genome v8, and the corresponding SNP loci 52319139, 52319152, and 52319160 in the reference genome v11 were also within 21 base pairs. However, there were differences in distances between reference genome v8 and v11 for the rest of the SNP loci. These disparities arise because the v11 reference genome is larger, incorporating additional nucleotides between these SNP loci (Table 7).

**Table 7:** Mapping of the ten stable SNPs on lettuce chromosomes 2, 4 and 7 from reference genome v8 to v11.

Chromosome	Reference genome v8		Reference genome v11	
	SNP location	Distance (bp) from adjacent SNP^a^	SNP location	Distance (bp) from adjacent SNP
2	109644094	-	122479112	-
4	38095762	248444472	41629889	270183372
4	286540234	88150795	311813261	95474789
4	374691029	-	407288050	-
7	50257083	13	52319139	13
7	50257096	8	52319152	8
7	50257104	295,304	52319160	294,918
7	50552408	956,664	52614078	962,326
7	51509072	107,358	53576404	106,331
7	51616430	-	53682735	-

a, SNP loci distance, e.g., 50257096–50257083 = 13 bp.

Reference genome size of v8 = 2.4 Gb and v11 = 2.6 Gb.

### Favorable allele effect

The allele effect of each of the stable SNPs was determined for the two traits GR5d_S and SSI_GR5d by comparing the average trait values of the two types of homozygous genotypes (homozygous for one allele or the other) at the SNP locus and the significance of the difference was determined by the Kruskal-Wallis test. Genotypes homozygous for minor allele and major allele were designated as minor and major genotypes, respectively (Table 8). The SNP *Ls*v8Lg2-109644094 had 401 major genotypes, 44 heterozygotes, but no minor genotype. The SNP *Lsv*8Lg4-38095762 had a minor allele frequency of 0.08, and the average GR5d_S for the minor and major genotypes were 95% and 75%, respectively, and the average GR5d_S of the minor genotype was significantly (*P* <0.001) higher (by 20%) than that of the major genotype based on Kruskal-Wallis test (Table 8). Thus, the minor allele of this SNP was favorable. Similarly, the average SSI_GR5d for the minor and major genotypes of this SNP were 0.19 and 1.06, respectively, and the average SSI_GR5d for the minor genotype was significantly (*P* <0.001) lower (by 0.87) than that of the major genotype (lower value is better for SSI). Based on similar comparisons, the minor alleles were favorable for each of the six SNPs on chromosome 7, while the major alleles were favorable for the SNPs *Lsv*8Lg4-286540234 and *Lsv*8Lg4-374691029 (Table 8).

**Table 8:** Minor/major allele contributions to the two salt-tolerance related traits GR5d_S and SSI_GR5d in lettuce.

	Major genotype		Minor genotype		Minor allele		GR5d_S		SSI_GR5d	
SNP	Type	Number	Type	Number	Allele	Freq^a^	Major genotype average (%)	Minor genotype average (%)	Major genotype average	Minor genotype average
*Lsv*8Lg2-109644094	CC	401	TT	0	T	0.05	79	-	0.88	-
*Lsv*8Lg4-38095762	GG	406	AA	31	A	0.08	75	95***	1.06	0.19***
*Lsv*8Lg4-286540234	CC	351	TT	65	T	0.18	79***	61	0.88***	1.69
*Lsv*8Lg4-374691029	AA	266	TT	165	T	0.39	84***	65	0.67***	1.52
*Ls*v8Lg7-50257083	CC	275	TT	140	T	0.34	71	88***	1.25	0.48***
*Ls*v8Lg7-50257096	GG	275	AA	140	A	0.34	71	88***	1.25	0.48***
*Ls*v8Lg7-50257104	CC	275	TT	140	T	0.34	71	88***	1.25	0.48***
*Ls*v8Lg7-50552408	AA	264	CC	151	C	0.37	72	86***	1.21	0.61***
*Ls*v8Lg7-51509072	AA	269	GG	146	G	0.35	72	85***	1.19	0.62***
*Ls*v8Lg7-51616430	CC	268	TT	147	T	0.36	72	85***	1.19	0.64***

***, indicates significantly (*P* < 0.001) different from the corresponding major/minor genotype value based on Kruskal-Wallis test.

^a^ including heterozygotes

### Crossing over and recombination of SNP alleles

The six significant SNPs on chromosome 7 were located within a 1.36 Mb region in the reference genome v8. The LD (r^2^ and D´) among these six SNPs are presented in S8 Table. Crossing over among these six SNPs were also manually evaluated (S9 Table). However, due to heterozygosity, crossing over could not be evaluated for few accessions. The three SNP loci 50257083, 50257096 and 50257104 were within a 21 base pair length in the sequence, and both the r2 and the D´ values of the three LDs among these three SNPs were 1, indicating the SNP pairs are in complete LD. However, one crossover event between SNP loci 50257096 and 50257104 was identified from the accession BM_GBS_25, where SNP locus 50257104 was heterozygous (S9 Table). Out of the 445 accessions in our study, 19 accessions were at heterozygous state for the SNP loci 50257083, 50257096 and 50257104, thus it was not possible to determine if there was any crossing over among the three SNPs for these 19 accessions. This finding indicated that these three SNPs were predominantly inherited as a haplotype, and the allelic effect of each of these SNPs was the same (Table 8). The distance between the SNP loci 50257104 and 50552408 was ~295.3 Kb and four crossovers were observed between these two SNP loci. The distance between SNP loci 50552408 and 51509072 was ~956.7 Kb and eight crossovers were observed between these two SNP loci. There was one crossover between the SNP loci 51509072 and 51616430 and the distance was ~ 107.4 Kb between these two loci. Since there were 445 accessions in the present study, the crossover rate was 2 to 4-fold greater than average crossover rate among the SNP loci 50257104, 50552408, 51509072 and 51616430. Although the D´ values for few of these SNP pairs were 1, the r2 values were less than 1, indicating occurrence of some recombination events among these SNP loci (S8 Table). The genotypes at the six SNP loci of the seven wild type (*L*. *serriola*) accessions in the present study are given in S10 Table. Five of the seven accessions had genotype ‘CGCCGT’, while the genotypes of the other two were ‘TATCGT’ and ‘YRYCGT’, where the alleles for the two heterozygous loci were Y = C/T and R = A/G. Out of the 445 accessions in the present study, only two other accessions, a primitive type, and a leaf type, had the genotype ‘CGCCGT’. Other than the seven wild type accessions, there were 21 non-cultivated type and 417 cultivated type of accessions and 262 of these accessions had the genotype ‘CGCAAC’ (major alleles) and 138 accessions had the genotype ‘TATCGT’ (minor alleles) for these six SNP loci (S10 Table). Although the number of *L*. *serriola* accessions was limited in this study, based on the available information, it appears that the alternate alleles at all six SNP loci may have evolved from *L*. *serriola* and have become common alleles in the cultivated types of lettuce (S9 and S10 Tables). Analyses of the recombination patterns among these six SNPs indicated that the minor/major alleles were in coupling phase linkage (S9 Table).

### Identification of putative candidate genes

A total of 25 salt tolerance-related and several other putative/predicted candidate genes were identified through NCBI BLAST search within 100 Kb upstream and downstream of each of the 10 significant SNPs identified in the present study. A list of these 25 putative/predicted salt tolerance-related genes along with brief description of their function/expression is presented in Table 9. The 25 genes were located over all lettuce chromosomes except chromosome 1, with seven genes on chromosome 7, and one to three genes on the rest of the chromosomes, while chromosome location of two of these genes were unknown. Nine of the genes had 100 percent sequence identity within the BLAST search, while the rest of the genes displayed sequence identity ranging from 83 to 98 percent. Four of the identified candidate genes encode the four transcription factors bHLH041, enhanced ethylene response protein 5 (EER5), MYB36, and bZIP from four major transcription factor families in plants.

**Table 9:** List of 25 salt tolerance-related candidate genes and their functions/expressions.

Name	Gene ID	Protein ID	Chr^a^	% Identity	Gene Function/expression	Reference
*Lactuca sativa* calcium-dependent protein kinase 24	111888214	XP_023740119.1	2	100	That several calcium dependent protein kinases (CPKs) function as positive regulators of salt tolerance in Arabidopsis have been well documented. The role of CPKs in salt tolerance have also been reported in other crops including rice and cotton.	[58–60]
*L*. *sativa* probably inactive leucine-rich repeat receptor-like protein kinase At3g28040	111887980	XP_023739879.1	2	100	Leucine-rich repeat receptor-like kinases (LRR-RLKs) have been reported to be associated with plant abiotic stress tolerance. An LRR-RLK, OsSTLK, overexpression conferred improved salt tolerance in rice. Although the encoded RLK by At3g28040 was predicted to be catalytically inactive, a recent study indicated its physical interaction with the *Arabidopsis* membrane-associated transcription factor ANAC089.	[61–63]
*L*. *sativa* serine carboxypeptidase-like 11	111889161	XP_052624522.1	2	95	The serine carboxypeptidase-like protein (SCPL) family plays a vital role in stress response including salinity tolerance	[64]
*L*. *sativa* glutathione S-transferase T2-like	111886501	XP_023738522.1	3	95	Overexpression of glutathione S-transferase gene from *Suaeda salsa* increases salt tolerance of Arabidopsis. Overexpression of a glutathione S-transferase gene, GsGST, from *Glycine soja* enhanced drought and salt tolerance in transgenic tobacco.	[65, 66]
*L*. *sativa* glutamate cysteine ligase chloroplastic	111893234	XP_023745068.1	3	89	Glutamate cysteine ligase has an important role in salt tolerance in plants.	[67, 68]
*L*. *sativa* F-box/ kelch-repeat protein At1g22040	111905221	Unknown	3	87	Response of F-box family genes to salt stress has been reported in Medicago trancatula and soybean.	[69, 70]
*L*. *sativa* non-specific lipid transfer protein GPI-anchored 30	111921808	XP_023773159.1	4	100	Plant non-specific lipid transfer proteins (nsLTPs) are small, basic proteins in higher plants. Both TaLTP40 and TaLTP75 from *Triticum aestivum* overexpressed in *Arabidopsis* revealed salt tolerance in comparison to wild type. GPI-anchored lipid transfer proteins have been reported to be involved in development of suberin in seed coat and thus limits salt uptake during seed germination.	[71–73]
*L*. *sativa* plant UBX domain-containing protein	111921696	XP_023773053.1	4	100	UBX domain-containing protein in rice was significantly upregulated under salt stress.	[74]
*L*. *sativa* callose synthase 4-like	122194482	XP_042758011.1	5	100	Callose is a key regulator of plasmodesmata transport in response to osmotic and salinity stress. Callose synthase gene was upregulated under salt stress in jojoba leaves.	[75, 76]
*L*. *sativa* basic leucine zipper 19	111910129	XP_023761682.2	5	87	Plant basic leucine zipper (bZIP) transcription factor is involved in many biological processes and plays an important role in tolerance to abiotic stresses including salt tolerance.	[77]
*L*. *sativa* homeobox protein knotted-1-like 11	111914121	XP_042751936.1	6	83	It has been shown that transcription factors encoded by knotted homeobox (KNOX) gene family play important roles in salt tolerance and development in cotton.	[78]
L. sativa cytokinin dehydrogenase 3	111905559	XP_052621353.1	7	100	Degrades cytokinins and the reduced levels of cytokinins give enhanced salt and drought tolerance in plants.	[79, 80]
*L*. *sativa* enhanced ethylene response protein 5, EER5	111905650	XP_023757113.1	7	100	EER5 protein interacts with other proteins during or following ethylene signaling and may have a role in salt stress tolerance in plants.	[81–83]
*L*. *sativa* 1-aminocyclopropane carboxylate oxidase 1 (ACO)	111905714	XP_023757208.1	7	100	T3 generation transgenic rice with barley ACO gene exhibited salinity tolerance.	[84]
*L*. *sativa* putative transcription factor bHLH041	111905512	XP_052621533.1	7	100	bHLH transcription factor is located upstream of the respiratory burst oxidase homolog (Rboh) promoter and overexpression of bHLH conferred greater salt tolerance in tobacco.	[85]
*L*. *sativa* peroxisomal fatty acid beta oxidation multifunctional protein AIM1	111884658	Unknown	7	95	Blocking fatty acid import into peroxisomes reduces reactive oxygen species accumulation and increases plant tolerance to salt stress, whereas increasing fatty acid flux into the β-oxidation pathway has opposite effects.	[86]
*L*. *sativa* transcription factor MYB36	111879582	XP_023731817.1	7	95	MYB transcription factor gene was upregulated in response to salt stress in wheat.	[87]
*L*. *sativa* pentatricopeptide repeat containing protein At5g46460	111880824	Unknown	7	85	Pentatricopeptide repeat (PPR)-containing proteins have been shown to be involved in the response to biotic and abiotic stresses in Arabidopsis, specifically improving salinity tolerance in Arabidopsis.	[88, 89]
*L*. *sativa* 60S ribosomal protein L6, mitochondrial.	111915146	XP_023766596.1	8	92	Recent studies have elucidated the role of ribosomal protein large (RPL) gene in salt tolerance in rice and cotton.	[90, 91]
*L*. *sativa* transcription elongation factor SPT-like	128127619	XP_052622223.1	8	90	A recent study demonstrated that the transcription elongation factor AtSPT4-2 positively modulates salt tolerance in *A*. *thaliana* by maintaining ion homeostasis and regulating stress responsive genes.	[92]
*L*. *sativa* serine/threonine protein kinase Atpk2/Atpk19	122195047	A0A9R1XPR2	8	97	Downstream effector of TOR signaling pathway. May be involved in adaptation of plant to cold or high-salt conditions.	https://www.uniprot.org/uniprotkb/Q39030/entry
*L*. *sativa* xylan O-acetyl transferase	111896847	XP_023748597.1	9	98	Xylan O-acetyl transferase has been shown to be involved in salt tolerance in Arabidopsis.	[93, 94]
*L*. *sativa* rab GTPase-activating protein 22	111912741	XP_023764246.1	9	92	The Ras-associated binding (Rab) family of small GTPases regulates intracellular membrane trafficking.	[95]
*L*. *sativa* cysteine-rich receptor like kinases	Unknown	AGM34085.1	Unknown	96	Cysteine-rich receptor-like kinases (CRKs) are evolutionarily conserved receptor-like kinases and many of them have been shown to regulate plant immunity and abiotic stress tolerance including salt tolerance.	[96]
*L sativa* 9-cis-epoxycarotenoid dioxygenase 4	111914373	AGM34083.1	Unknown	86	9-cis-epoxycarotenoid dioxygenase is an enzyme needed for abscisic acid (ABA) biosynthesis, and that it regulates salt and water stress tolerance in rice has been elucidated by Huang et al.	[97]

^a^Chromosome #

Distances of these salt-tolerance related genes from the identified SNPs are presented in Table 10. Two of these genes: the *L*. *sativa* cytokinin dehydrogenase 3 gene and the transcription factor EER5 encoding gene were located in the 1.36 Mb region on chromosome 7, harboring the six closely linked SNPs associated with the QTL qSTgs7.1. The *L*. *sativa* putative transcription factor bHLH041encoding gene was located 58.4 Kb downstream of the SNP *Ls*v8Lg7-50257083, while the *L*. *sativa* 1-aminocyclopropane carboxylate oxidase 1 gene was located 77.1 Kb upstream of the SNP *Ls*v8Lg7-51616430. Three other candidate genes were located within <100 Kb from three of the identified SNPs (Table 10).

**Table 10:** Candidate gene locations relative to identified SNPs.

Gene name	Chr	Gene location range in reference genome v8	Distance from closest SNP
*Lactuca sativa* calcium-dependent protein kinase 24	2	109726740–109729918	82.6 Kb upstream of *Ls*v8Lg2-109644094
*L*. *sativa* probably inactive leucine-rich repeat receptor-like protein kinase At3g28040	2	109677918–109681399	33.8 Kb upstream of *Ls*v8Lg2-109644094
*L*. *sativa* serine carboxypeptidase-like 11	2	171095870–171119310	61.5 Mb upstream of *Ls*v8Lg2-109644094
*L*. *sativa* non-specific lipid transfer protein GPI-anchored 30	4	374783765–374784564	92.7 Kb upstream of *Ls*v8Lg4-374691029
*L*. *sativa* plant UBX domain-containing protein	4	374778164–374780154	87.1 Kb upstream of *Ls*v8Lg4-374691029
*L*. *sativa* cytokinin dehydrogenase 3	7	50542933–50545632^a^	6.8 Kb downstream of *Ls*v8Lg7-50552408
*L*. *sativa* enhanced ethylene response protein 5, EER5	7	51465321–51468482^a^	40.6 Kb downstream of *Ls*v8Lg7-51509072
*L*. *sativa* 1-aminocyclopropane carboxylate oxidase 1 (ACO)	7	51693544–51694961	77.1 Kb upstream of *Ls*v8Lg7-51616430
*L*. *sativa* putative transcription factor bHLH041	7	50194212–50198695	58.4 Kb downstream of *Ls*v8Lg7-50257083
*L*. *sativa* peroxisomal fatty acid beta oxidation multifunctional protein AIM1	7	29827369–29840138	20.4 Mb downstream of Lsv8Lg7-50257083
*L*. *sativa* transcription factor MYB36	7	74814007–74818667	23.2 Mb upstream of Lsv8Lg7-51616430
*L*. *sativa* pentatricopeptide repeat containing protein At5g46460	7	36535683–36539744	13.7 Mb downstream of Lsv8Lg7-50257083

^a^, *L*. *sativa* cytokinin dehydrogenase 3 and the EER5 transcription factor protein genes fall within the 1.36 Mb region on chromosome 7 harboring the six closely linked SNPs signaling the QTL qSTgs7.1.

## Discussion

Breeding lettuce varieties that can germinate and establish in salt-affected soil is essential since lettuce seeds are planted half an inch deep in soil where salinity levels are often the highest in salinity-affected growing regions. In recent years, GWAS has become a very useful tool for identifying markers associated with traits that can be effectively used by plant breeders for developing new and improved crop varieties. In the present research, we conducted a GWAS to identify SNP markers associated with salt tolerance at the seed germination stage in lettuce. A soil salinity level of 100 mM NaCl is about as high as most crop plants tolerate without significant reduction in growth and yield [98] and an earlier study on lettuce seed germination in our laboratory (unpublished data) with 70, 80, and 100 mM NaCl stress conditions indicated highest genetic variability among the tested entries with 100 mM NaCl stress. Therefore, in the present study, we used a salt stress condition of 100 mM NaCl. Through this research, we successfully identified 10 highly significant SNPs associated with five QTLs for salt tolerance at the seed germination stage. GWAS has been successfully applied for discovering marker-trait associations for several traits in lettuce [29, 30–33]. However, to our knowledge, this is the first marker-trait association study of any kind on salt tolerance at the seed germination stage in lettuce and is the first report on genome-wide association study of salt tolerance in lettuce. GWAS for salt tolerance at the seed germination stage has been reported in rice, mungbean, flax and *Brassica napus* [34–37].

Some of the most important considerations in GWAS are: genetic diversity within the GWAS panel, sample size, trait diversity, linkage disequilibrium, and population structure [20, 99, 100]. To ensure sufficient genetic diversity in a GWAS panel, mixed populations containing subpopulations of different genetic backgrounds may be suitable, as a simulation study demonstrated that GWAS’ power with a mixed population was generally higher than that of with a separate population [99]. That diverse populations add power to GWAS results has also been shown in GWAS with human populations, where diverse populations facilitated the discovery of novel type 2 diabetes aetiological factors owing to their divergent allele frequency across populations [101]. An earlier study with 441 accessions from the same GWAS panel and with 400 common accessions as the present study found the GWAS panel to be diverse [31]. In that study, PCA clustered the four major horticultural types: butterhead, crisphead, leaf and romaine into separate clusters with some overlap of the butterhead and romaine types with leaf type [31]. Since 45 accessions in the present study were different from the earlier study [31], we also conducted PCA to assess the genetic diversity and population structure of the 445 accessions in the current study. We included the four major horticultural types as well as the stem and wild types of lettuce accessions in this PCA. The PCA revealed clustering of the lettuce accessions according to their horticultural types (Fig 2). Thus, our genetic diversity analysis through PCA confirmed the result of the earlier study [31] that our GWAS panel was diverse. This diversity among groups or subpopulations in our GWAS panel likely contributed to the identification of the 10 significant SNPs in our study.

In human populations, where hundreds of thousands to over a million SNPs are commonly used for GWAS, sample sizes typically exceed 1,000 [102], while in plant populations generally a lower number of SNPs are used, and many successful GWAS studies in plants have been conducted with a sample size of a few hundred [103]. Our GWAS panel consisted of 445 diverse lettuce accessions which is a higher number of accessions than commonly used for GWAS in plants [103]. In the present study, descriptive statistics and ANOVA indicated substantial phenotypic variation for the seed germination-related traits under salt stress (Table 1 and S2 Table). Broad-sense heritability estimates of these traits were high, indicating that most of the phenotypic variation was due to genetic factors (S2 Table).

LD is a measure of the correlation between alleles at different loci. Rapid decay of LD is favorable for association testing of candidate genes that are located near mapped QTLs and have functional relevance to trait variation [45]. In maize, LD generally decayed rapidly with distance (r^2^ <0.1 within 1.5 Kb), however, rates of decline were highly variable among genes [45]. In general, LD decay is slower in self-pollinated species like lettuce as compared to cross-pollinated species, where recombination rate is much higher [104]. The LD decay in our GWAS panel was comparable to other self-pollinated species and was suitable for GWAS. For example, LD decayed to half of its maximum at approximately 450–500 Kb and 500–550 Kb in *desi* and *kabuli* chickpeas, respectively [105]. LD decayed to half of its maximum at ~75 Kb and ~150 Kb, in wild and cultivated soybeans, respectively (106). In a separate study, LD decayed to half of its maximum at 60 Kb and 100 Kb, respectively, in wild and cultivated types of mungbeans [107]. The LD decay examples from soybean and mungbean above showed that the LD decays of the wild types were faster compared to the cultivated type [106, 107]. These results are in congruence with the result in the present study as we observed that the LD decay to half of its maximum for the 445 lettuce accessions (at 290.8 Kb) including 417 cultivated, seven wild, and 21 non-cultivated types was faster than that of the 417 cultivated type accessions (at 351.8 Kb). It should be noted here that the seven wild type accessions in our study were too few to conduct LD analysis and compute LD decay. In a previous study that included 400 common accessions to the present study, LD decay of the four major cultivated horticultural types were computed separately, resulting in LD decay to half of its maximum at 241 Kb, 349 Kb, 388 Kb and 746 Kb, respectively, for leaf, romaine, butterhead and crisphead types [31].

The 10 significant SNPs identified in the present study were the same for both the salt tolerance-related traits GR5_S and SSI_GR5d (Table 5). The two traits were essentially the same, however, the formula for computing SSI compares seed germination rate under salinity stress to germination rate under non-salinity or control (water) condition, thus it reflects the true genetic variability among the lettuce accessions under salt-stress and avoids the confounding effects of loss of seed viability due to seed age, dormancy, or seed being affected by disease. In the absence of such confounding effects, the results from SSI_GR5d and GR5d_S would be the same or similar. However, we used both the traits to compare the results, and because the results from GR5d_S (percent germination) is more interpretable and comprehensible than SSI_GR5d (an index), especially when comparing favorable allele effects using the significant SNPs and phenotypic data.

An earlier study [16], using a RIL population derived from a bi-parental cross, identified 9 QTLs for salt tolerance at the seedling stage in lettuce including a major QTL on chromosome 2 and one major and one minor QTLs located on chromosome 7. The QTL on chromosome 2 (reference genome, v8), mapped to ~ 23.2 Mb upstream of the SNP *Ls*v8Lg2-109644094/QTL qSTgs2.1, that we identified on chromosome 2. The major QTL on chromosome 7 mapped to ~ 33 Mb upstream of the end SNP *Ls*v8Lg7-51616430, while the minor QTL mapped to ~ 9.1 Mb downstream of the lead SNP *Ls*v8Lg7-50257083 of the QTL qSTgs7.1 in our study. Thus, the three QTLs were different from the QTLs identified in the present study. The locations of the four seedling-stage salt tolerance-related QTLs on lettuce chromosome 7, identified in a separate study [17] were also different as compared to the locations of the five QTLs in the present study. Thus, the five QTLs identified in our study are novel QTLs for salt tolerance in lettuce.

Minor alleles were favorable for all six significant SNP loci on chromosome 7 and for one SNP on chromosome 4, while major alleles were favorable for two SNPs on chromosome 4, for both the traits GR5d_S and SSI_GR5d. This favorable allele information will be useful for breeding lettuce for salinity tolerance. Favorable effect of minor allele for salt tolerance related SNPs/haplotype at the seed germination stage were reported in rice [34]. Evaluation of crossing over and recombination among the six SNP loci in the present study revealed that the three SNPs, *Ls*v8Lg7-50257083, *Ls*v8Lg7-50257096 and *Ls*v8Lg7-50257104 predominantly inherited as a haplotype, whereas there were 2- to 4-fold greater than average crossing over among the four SNPs, *Ls*v8Lg7-50257104, *Ls*v8Lg7-50552408, *Ls*v8Lg7-51509072, and *Ls*v8Lg7-51616430. Greater than average recombination may arise for the benefit of creating genetic diversity through genomic rearrangement along with selection for maintaining favorable gene recombination [108]. Evaluation of the recombination patterns among these six SNPs also indicated that the minor/major alleles in these SNP loci were in coupling phase linkage (S9 Table).

Employing five models across two software packages (GAPIT and TASSEL), the present study demonstrated the advantages of using multiple models and software for GWAS in identifying marker-trait associations. This approach strengthens confidence in the results, aligning with the GAPIT user manual, which recommends validating findings with other software like TASSEL. In the present study, we identified 25 candidate genes with known salt tolerance-related functions including four genes encoding transcription factors from four major transcription factor families in plants. Extensive literature search on the function and expression analysis of these genes revealed their important roles in salt tolerance and potential involvement in other abiotic stress tolerance such as drought and cold tolerance (Table 9). These findings highlight their potential for further validation and application in marker-assisted selection for salt tolerance in lettuce.

## Conclusion

Soil salinity is one of the most important abiotic stress factors limiting crop production globally. For successful germination and establishment in the salinity-affected growing region it is important to breed lettuce varieties with salt tolerance at the seed germination stage. To our knowledge, this is the first study on GWAS to identify genomic regions associated with salt tolerance in lettuce and the first marker-trait association study of any kind for salt tolerance at the seed germination stage in lettuce. Ten significant marker-trait associations representing five novel QTLs for salt tolerance at the seed germination stage were identified in the current GWAS. Favorable alleles for these SNPs were also identified. Based on these SNP locations, twenty-five salt-tolerance related candidate genes including four genes encoding for transcription factors from four major transcription factor families in plants were identified. Collectively the findings in the current study will help develop molecular markers related to salt tolerance at the seed germination stage and thereby help breeding improvement of lettuce with enhanced salt tolerance at the seed germination stage.

## Supporting information

S1 File Phenotypic data used in this study. (XLSX)

S1 Fig Chromosome-wise linkage disequilibrium (LD) and LD decay for the nine lettuce chromosomes. LD and LD decay involving 56,820 SNPs and 445 lettuce accessions. LD decayed to half of its maximum at 261664 bp, 199542 bp, 349541 bp, 403689 bp, 259715 bp, 89382 bp, 232815 bp, 332532 bp, and 328898 bp for the chromosomes 1, 2, 3, 4, 5, 6, 7, 8 and 9, respectively.
(DOCX)

S2 Fig Distribution of residuals for the trait germination rate under salt stress, 5 days post seeding (GR5d_S). Residuals distribution for the trait GR5d_S from GWAS using mixed linear model (MLM) in the TASSEL software package.
(TIF)

S3 Fig Distribution of residuals for the trait salinity susceptibility index, 5 days post seeding (SSI_GR5d). Residuals distribution for the trait SSI_GR5d from GWAS using mixed linear model (MLM) in the TASSEL software package.
(TIF)

S1 Table List of the 445 *Lactuca* accessions in the GWAS panel. (XLSX)

S2 Table Analysis of variance (mean squares), genetic and phenotypic variance, and broad-sense heritability. (DOCX)

S3 Table Bayesian information criterion (BIC)-based model selection results. (DOCX)

S4 Table Comparison of results with 0, 1, 2 and 3 PCs in the GWAS models for the trait GR5d_S. (XLSX)

S5 Table Comparison of results with 0, 1, 2 and 3 PCs in the GWAS models for the trait SSI_GR5d. (XLSX)

S6 Table Significant SNPs identified for the trait GR5d_S by the TASSEL software. (XLSX)

S7 Table Significant SNPs identified for the trait SSI_GR5d by the TASSEL software. (XLSX)

S8 Table Linkage disequilibrium (r2), and D’ between pairs of the six significant SNPs on chromosome 7. (XLSX)

S9 Table Evaluation of crossing over/recombination and coupling phase linkage. (XLSX)

S10 Table Seven *L*. *serriola* accession genotypes at the six significant SNP loci on chromosome 7. (XLSX)
