# Supplementary material for: Genome-wide association study of salt tolerance at the seed germination stage in lettuce
Source: PLoS One. 2024 Oct 18;19(10):e0308818. doi: 10.1371/journal.pone.0308818 (PMC11488735; doi:10.1371/journal.pone.0308818)
Supplement: S1 Fig — LD and LD decay involving 56,820 SNPs and 445 lettuce accessions. LD decayed to half of its maximum at 261664 bp, 199542 bp, 349541 bp, 403689 bp, 259715 bp, 89382 bp, 232815 bp, 332532 bp, and 328898 bp for the chromosomes 1, 2, 3, 4, 5, 6, 7, 8 and 9, respectively. (DOCX) [file pone.0308818.s002.docx]

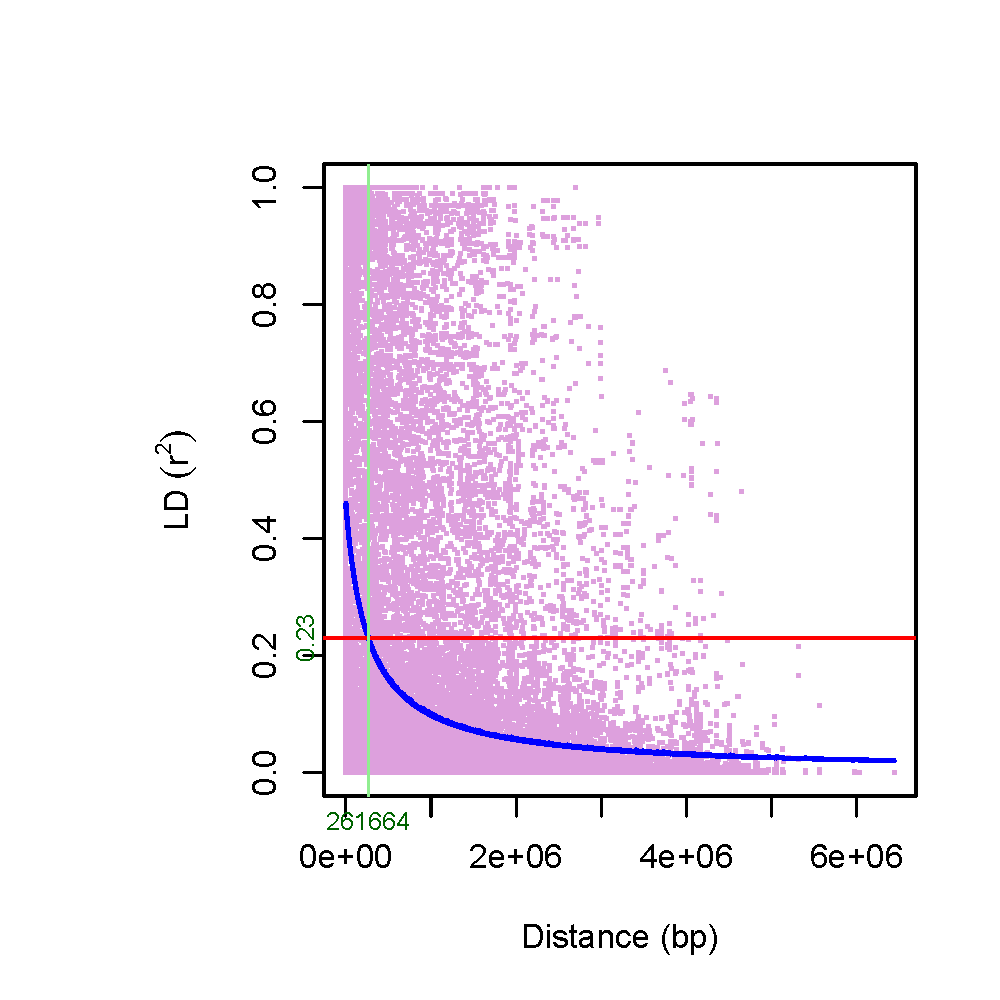

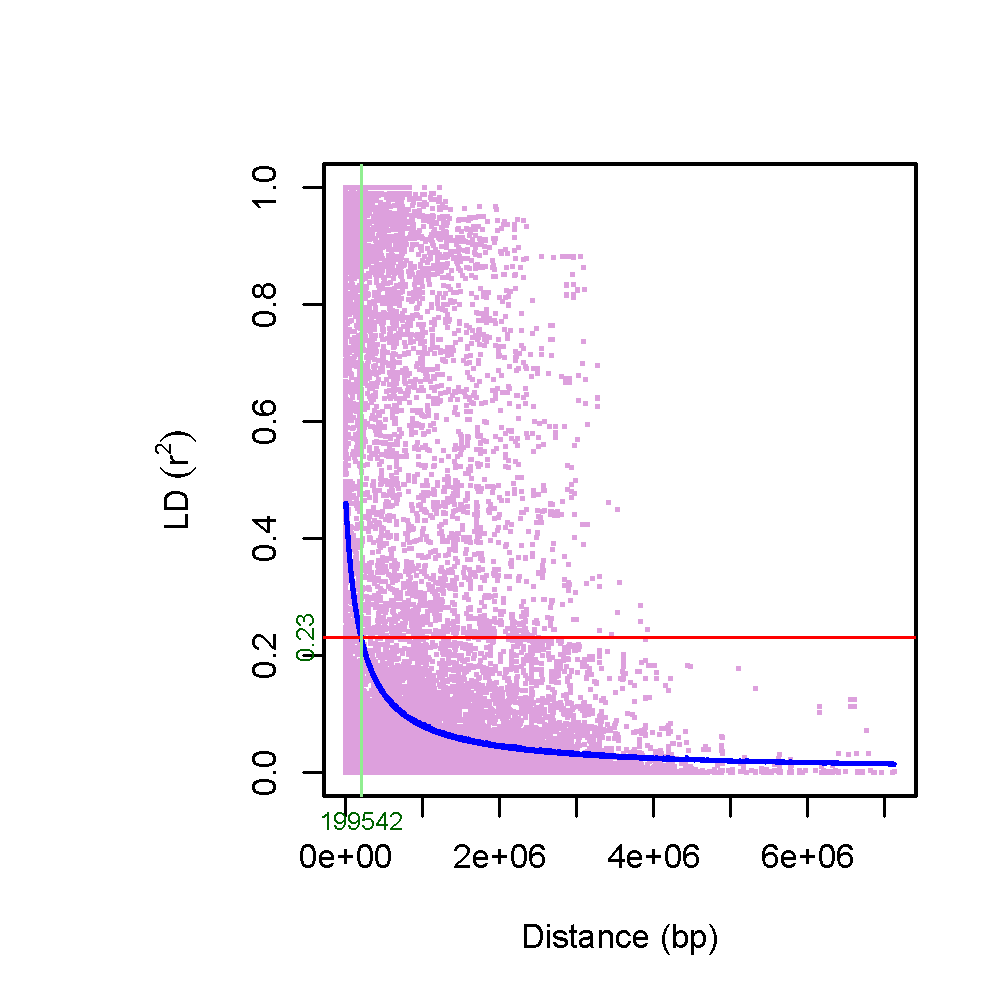

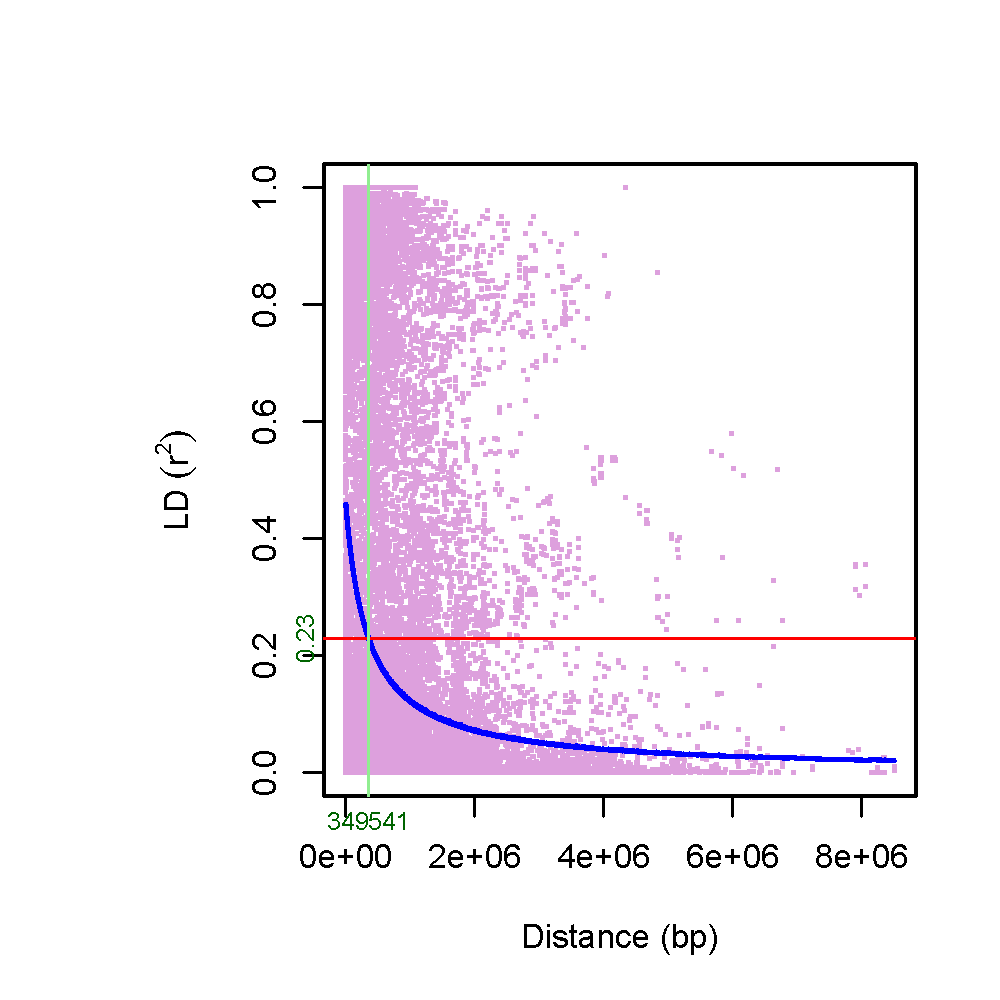


Chromosome 1 Chromosome 2 Chromosome 3


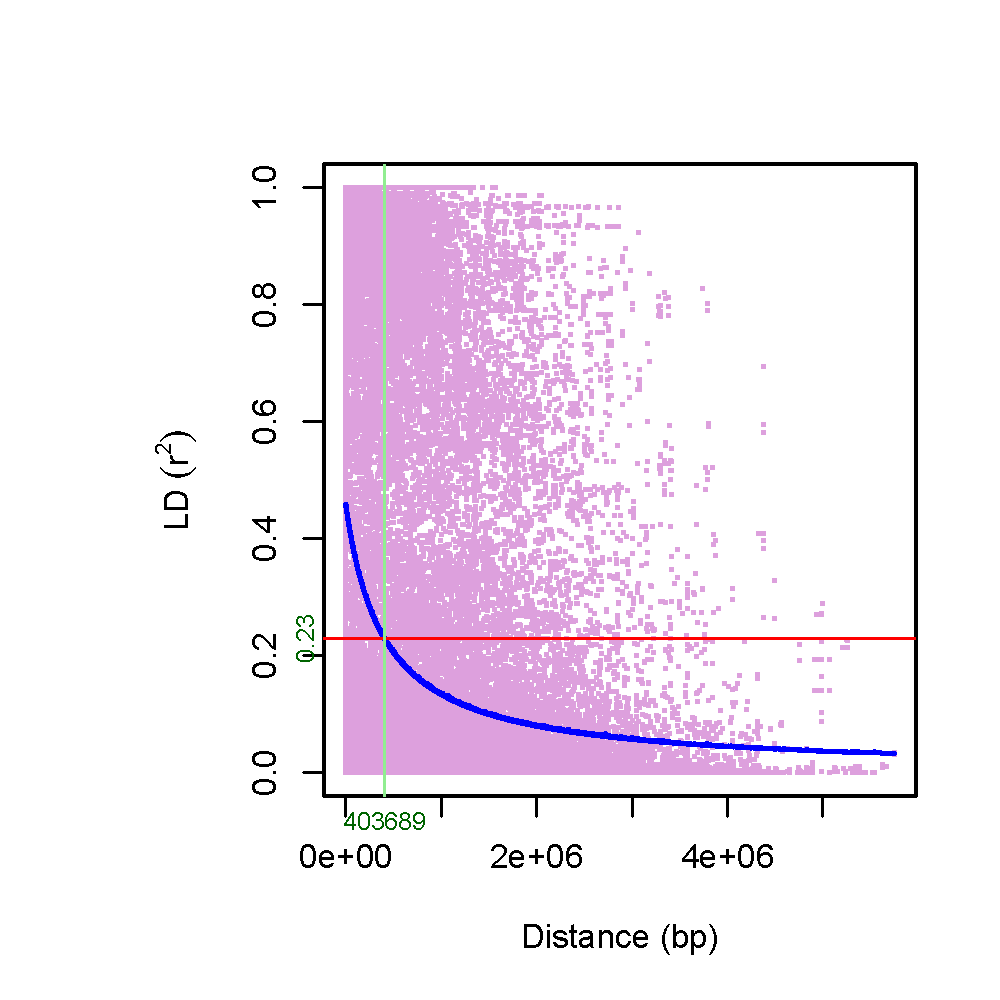

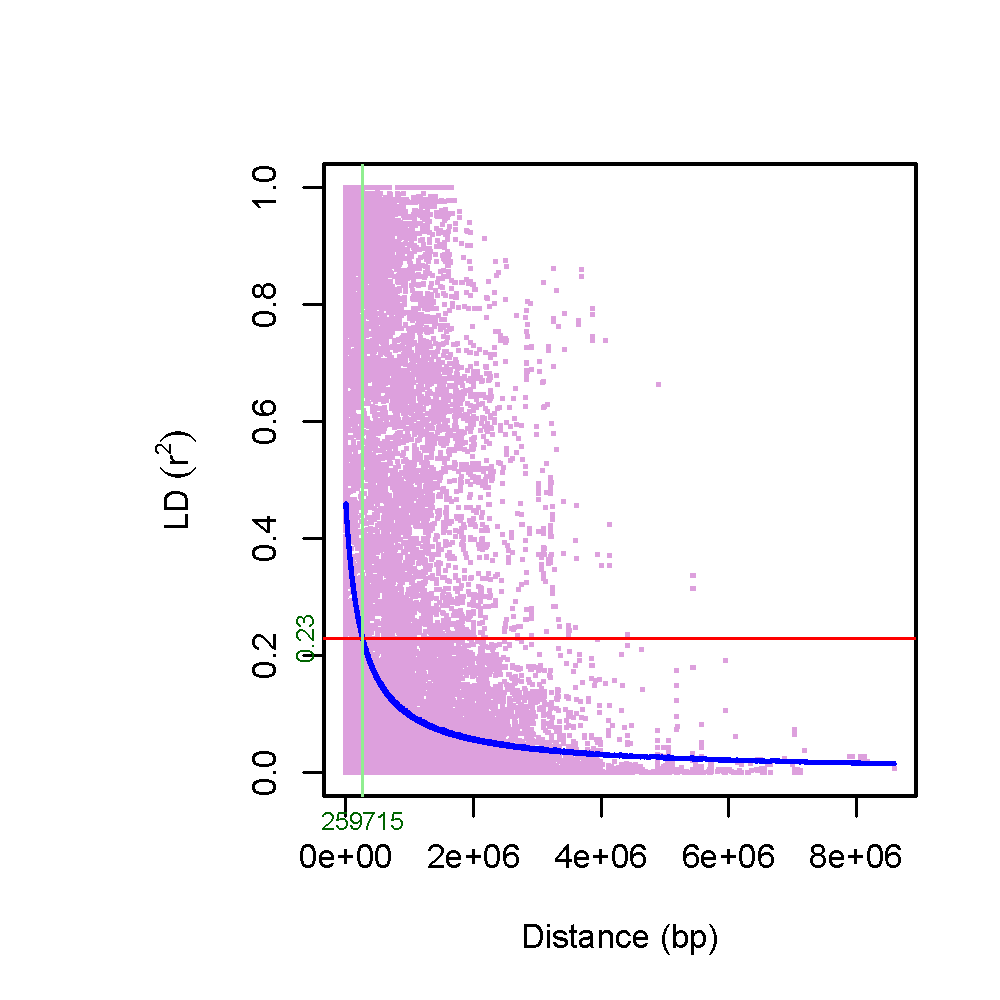

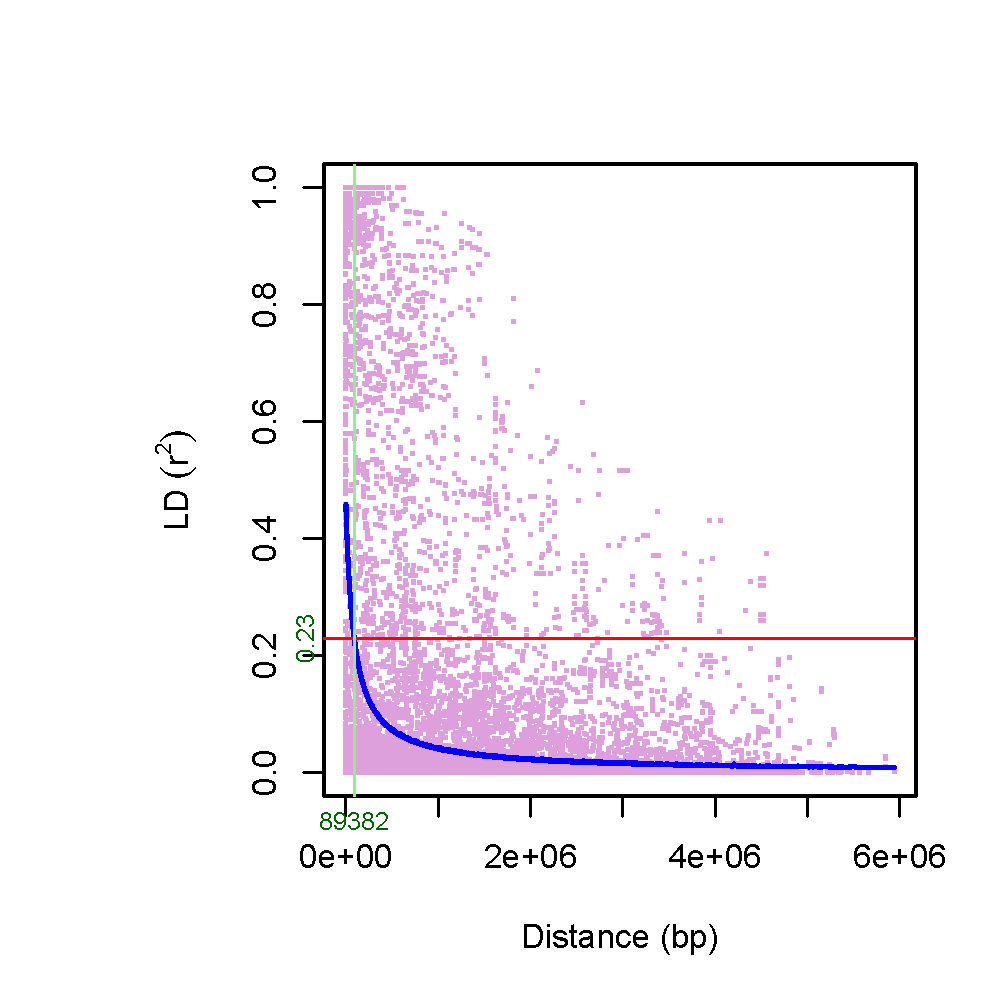


Chromosome 4 Chromosome 5 Chromosome 6


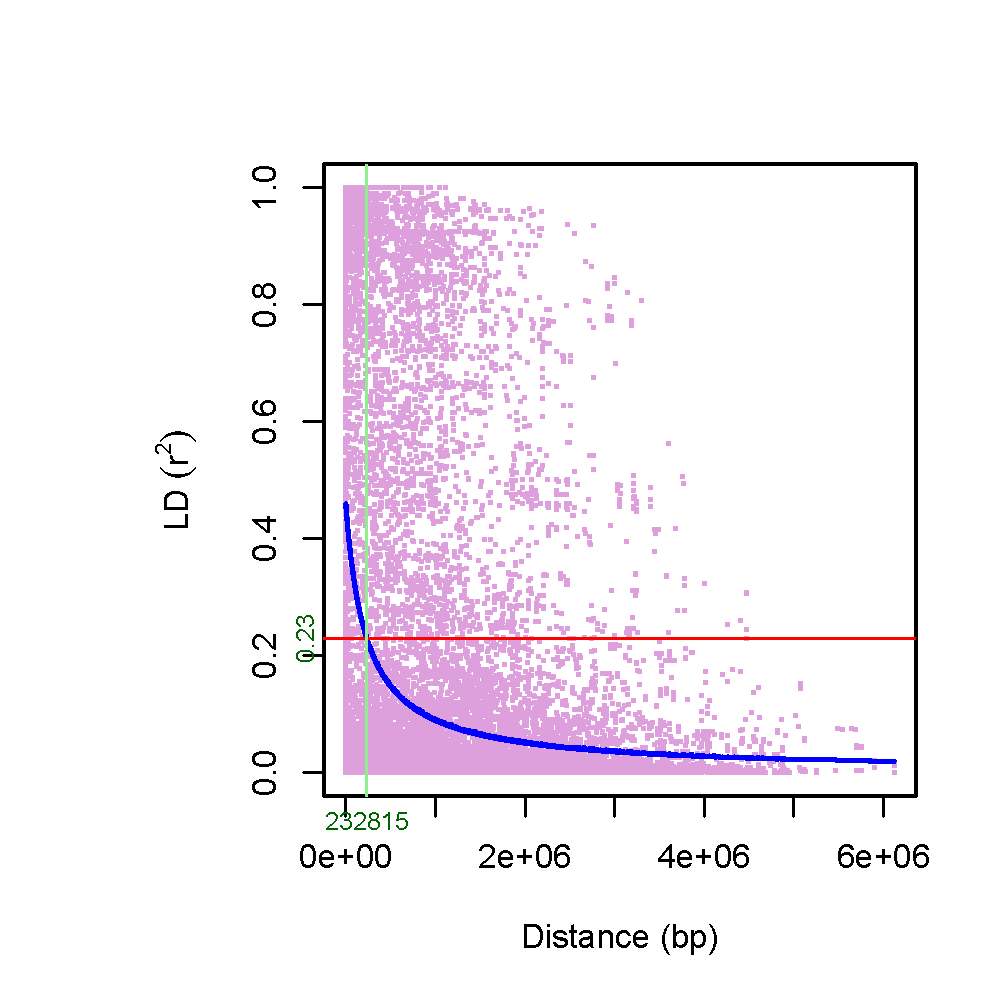

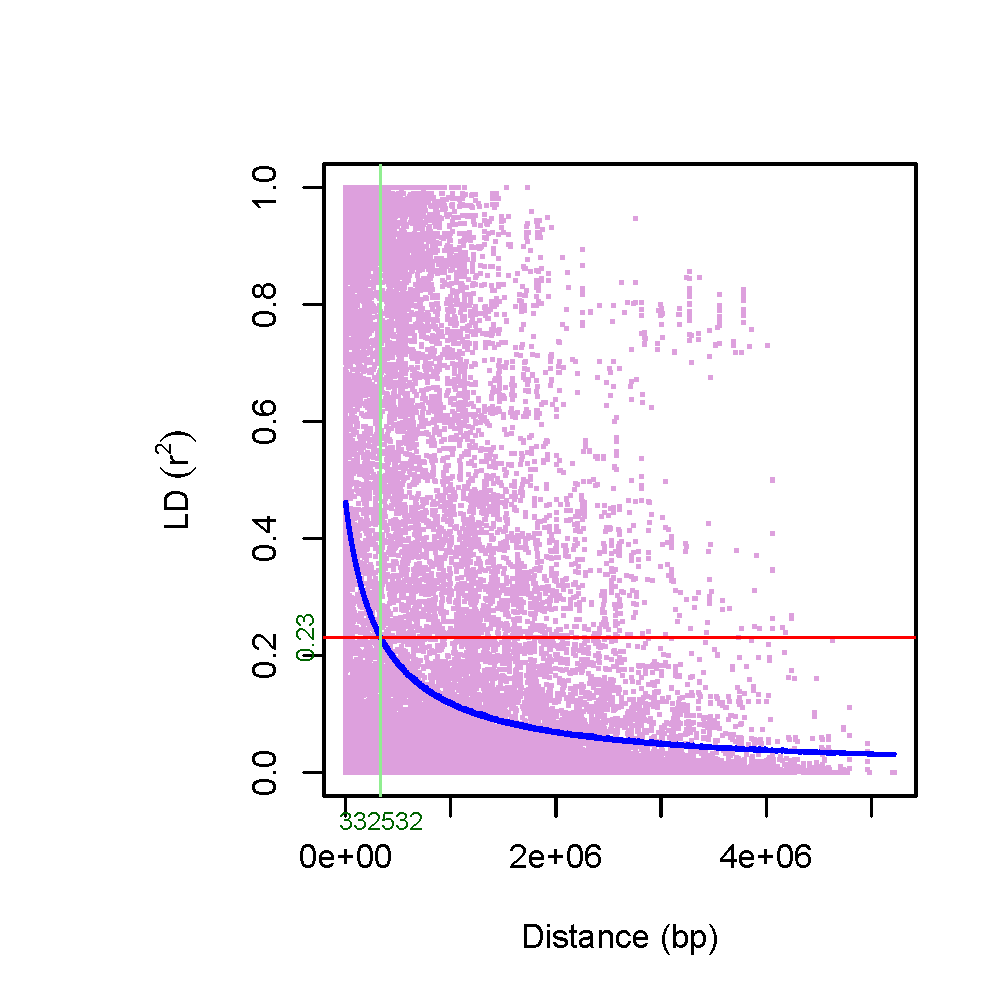

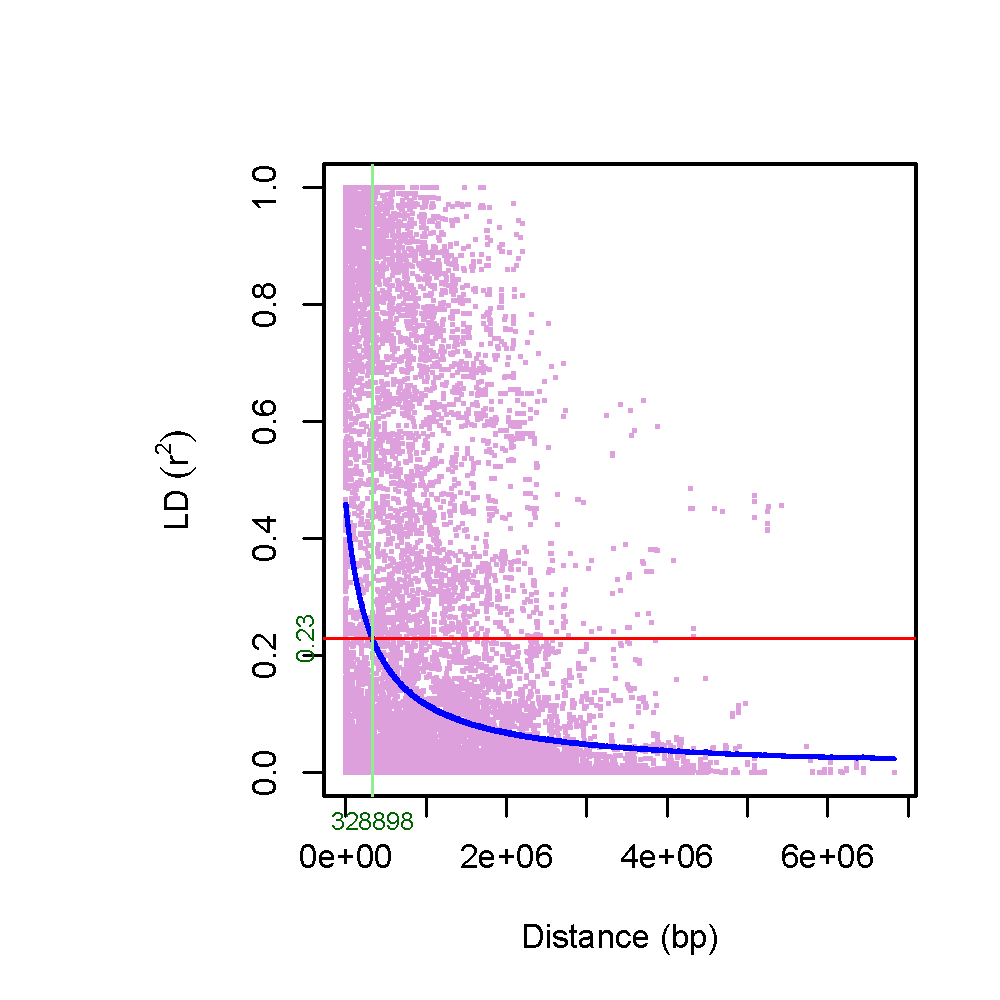


Chromosome 7 Chromosome 8 Chromosome 9
