# Supplementary figures and images for: Genome-wide association study of salt tolerance at the seed germination stage in lettuce
Source: PLoS One. 2024 Oct 18;19(10):e0308818. doi: 10.1371/journal.pone.0308818 (PMC11488735; doi:10.1371/journal.pone.0308818)

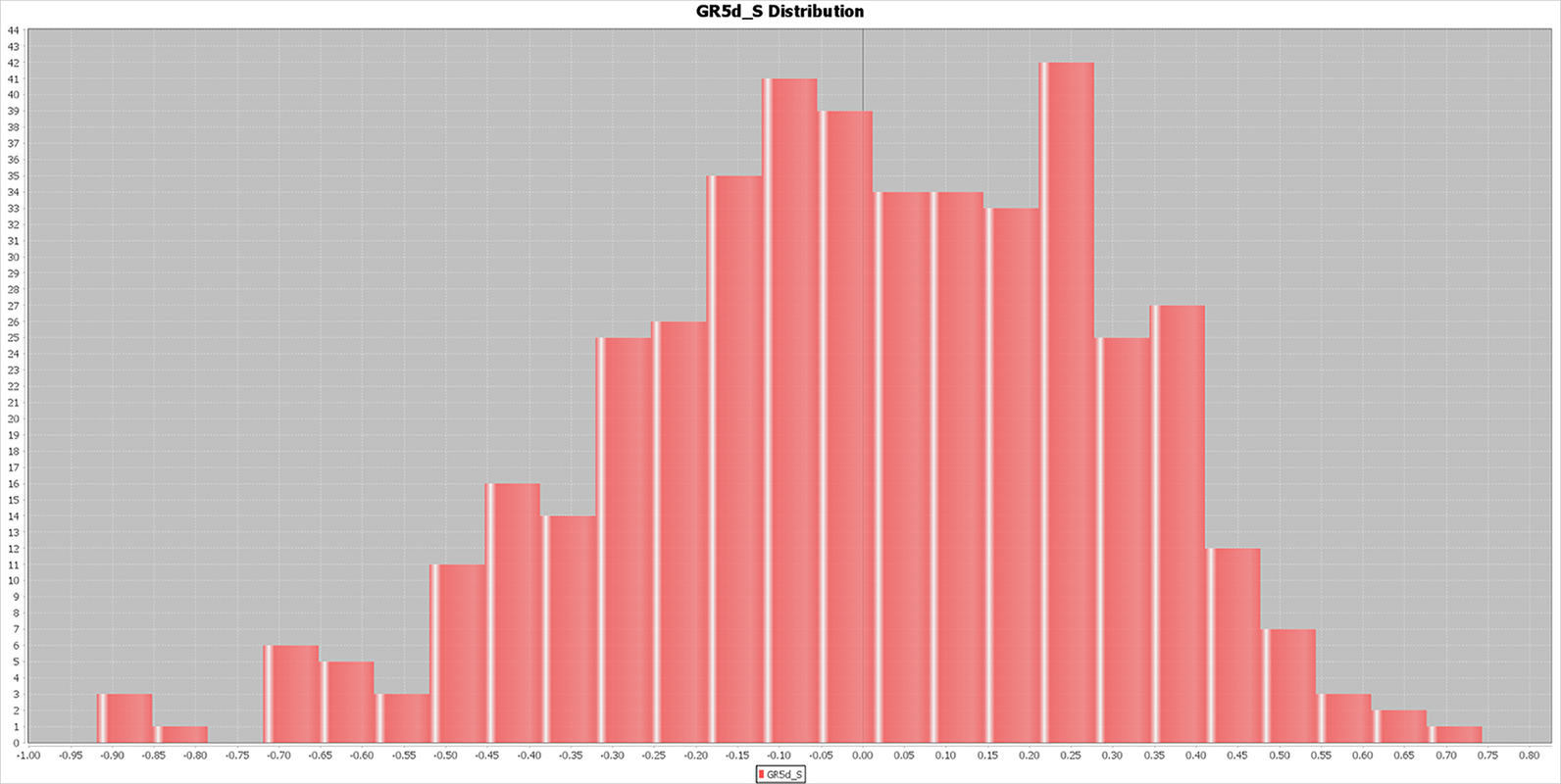

Supplement: S2 Fig — Residuals distribution for the trait GR5d_S from GWAS using mixed linear model (MLM) in the TASSEL software package. (TIF) [file pone.0308818.s003.tif]

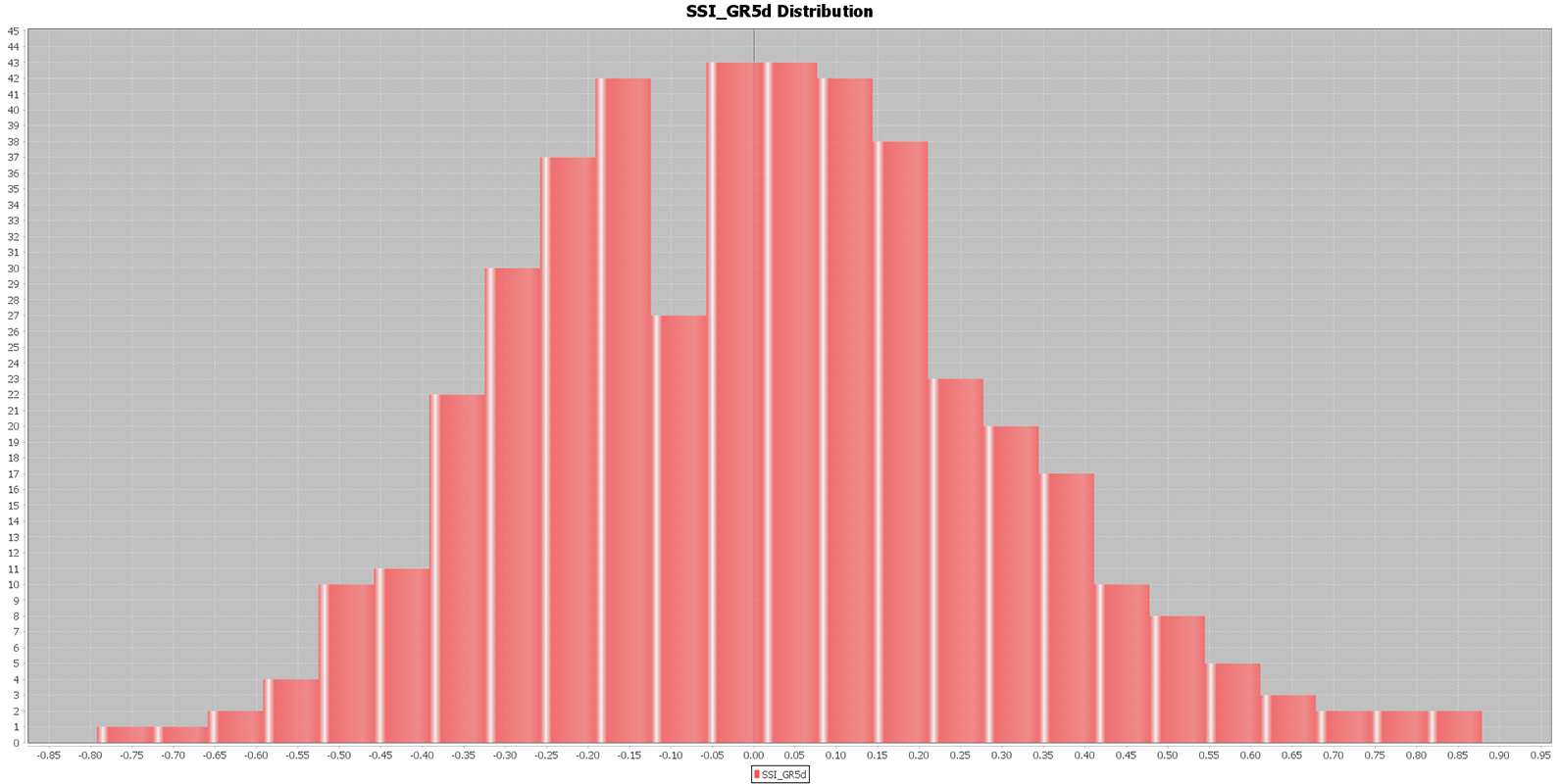

Supplement: S3 Fig — Residuals distribution for the trait SSI_GR5d from GWAS using mixed linear model (MLM) in the TASSEL software package. (TIF) [file pone.0308818.s004.tif]
