# Supplementary material for: Genome-wide association study of salt tolerance at the seed germination stage in lettuce
Source: PLoS One. 2024 Oct 18;19(10):e0308818. doi: 10.1371/journal.pone.0308818 (PMC11488735; doi:10.1371/journal.pone.0308818)
Supplement: S2 Table — (DOCX) [file pone.0308818.s006.docx]

**S2 Table. Analysis of variance (mean squares), genetic and phenotypic variance, and broad-sense heritability of seed germination related traits measured under salinity stress (100 mM NaCl) condition in lettuce.**

| **Source** | **Df** | **GR2d_S** | **GR5d_S** | **SSI_GR2d** | **SSI_GR5d** |
| --- | --- | --- | --- | --- | --- |
| Genotype/accession | 444 | 2.422*** | 2.206*** | 2.565*** | 2.375*** |
| Residuals | 890 | 0.113 | 0.121 | 0.169 | 0.191 |
| Genetic variance | - | 0.770 | 0.695 | 0.799 | 0.728 |
| Phenotypic variance | - | 0.808 | 0.735 | 0.855 | 0.792 |
| Heritability (broad-sense) | - | 0.95 | 0.95 | 0.93 | 0.92 |

***, significant at 0.001 level of probability.

GR2d_S: germination rate (GR) under salt stress, 2 days post seeding; GR5d_S: GR under salt stress, 5 days post seeding.

SSI_GR2d: salinity susceptibility index (SSI), 2 days post seeding; SSI_GR5d: SSI, 5 days post seeding.
