# Supplementary material for: Genome-wide association study of salt tolerance at the seed germination stage in lettuce
Source: PLoS One. 2024 Oct 18;19(10):e0308818. doi: 10.1371/journal.pone.0308818 (PMC11488735; doi:10.1371/journal.pone.0308818)
Supplement: S3 Table — (DOCX) [file pone.0308818.s007.docx]

**S3 Table. Bayesian information criterion (BIC)-based model selection results. The row with the largest BIC value has the optimal number of principal components to include in the GWAS models.**

| Number of principal components (PCs) | BIC | log Likelihood Function Value |
| --- | --- | --- |
| Trait – GR5d_S | | |
| 0 | -527.51 | -518.36 |
| 1 | -530.50 | -518.31 |
| 2 | -533.35 | -518.10 |
| 3 | -536.31 | -518.01 |
| Trait – SSI_GR5d | | |
| 0 | -544.00 | -534.86 |
| 1 | -546.99 | -534.80 |
| 2 | -549.87 | -534.62 |
| 3 | -552.84 | -534.55 |
